# Intestinal flora and pregnancy complications: Current insights and future prospects

**DOI:** 10.1002/imt2.167

**Published:** 2024-01-22

**Authors:** Zhenyu Tian, Xinjie Zhang, Guixiang Yao, Jiajia Jin, Tongxue Zhang, Chunhua Sun, Zhe Wang, Qunye Zhang

**Affiliations:** ^1^ National Key Laboratory for Innovation and Transformation of Luobing Theory; The Key Laboratory of Cardiovascular Remodeling and Function Research, Chinese Ministry of Education, Chinese National Health Commission and Chinese Academy of Medical Sciences; Department of Cardiology Qilu Hospital of Shandong University Jinan China; ^2^ Department of Biology University College London London UK; ^3^ Department of Health Management Center, Qilu Hospital, Cheeloo College of Medicine Shandong University Jinan China; ^4^ Department of Geriatrics Shandong Provincial Hospital Affiliated to Shandong First Medical University Jinan China; ^5^ Cardiovascular Disease Research Center of Shandong First Medical University Central Hospital Affiliated to Shandong First Medical University Jinan China

**Keywords:** gestational diabetes mellitus, gut microbiota, in utero colonization, microecological therapy, pre‐eclampsia, pregnancy complications, sterile womb

## Abstract

Numerous studies have demonstrated the pivotal roles of intestinal microbiota in many physiopathological processes through complex interactions with the host. As a unique period in a woman's lifespan, pregnancy is characterized by changes in hormones, immunity, and metabolism. The gut microbiota also changes during this period and plays a crucial role in maintaining a healthy pregnancy. Consequently, anomalies in the composition and function of the gut microbiota, namely, gut microbiota dysbiosis, can predispose individuals to various pregnancy complications, posing substantial risks to both maternal and neonatal health. However, there are still many controversies in this field, such as “sterile womb” versus “in utero colonization.” Therefore, a thorough understanding of the roles and mechanisms of gut microbiota in pregnancy and its complications is essential to safeguard the health of both mother and child. This review provides a comprehensive overview of the changes in gut microbiota during pregnancy, its abnormalities in common pregnancy complications, and potential etiological implications. It also explores the potential of gut microbiota in diagnosing and treating pregnancy complications and examines the possibility of gut‐derived bacteria residing in the uterus/placenta. Our aim is to expand knowledge in maternal and infant health from the gut microbiota perspective, aiding in developing new preventive and therapeutic strategies for pregnancy complications based on intestinal microecology.

## Highlights

- Pregnancy complications critically affect maternal and child health, necessitating urgent research and therapeutic strategies to reduce health and socioeconomic impacts.
- Gut microbiota dysbiosis in patients with various pregnancy complications acts as both a causal factor and a contributor to these conditions.
- Gut microbiota‐derived metabolites are involved in various pathophysiological pathways closely related to the pathogenesis of pregnancy complications, including intestinal barrier permeability, inflammatory responses, and glucose and lipid metabolism.
- Emerging therapeutic strategies based on gut microbes show potential in treating pregnancy complications, yet there is a lack of evidence from randomized controlled trials (RCTs) to substantiate this approach.

## Graphical abstract

Gut microbiota is crucial for a healthy pregnancy, being affected by factors such as hormones, diet, obesity, nicotine, antibiotics, and microplastics. This article emphasizes the impact of gut microbiome changes on pregnancy complications and outcomes, delving into the underlying mechanisms and potential gut microbe‐based therapies. Additionally, it explores the contentious issue of bacterial presence in the uterine environment. FGR, fetal growth restriction; HT, hypothyroidism in pregnancy; ICP, intrahepatic cholestasis of pregnancy; PE, pre‐eclampsia; GDM, gestational diabetes mellitus; PROM, premature rupture of membranes; PTB, preterm birth.

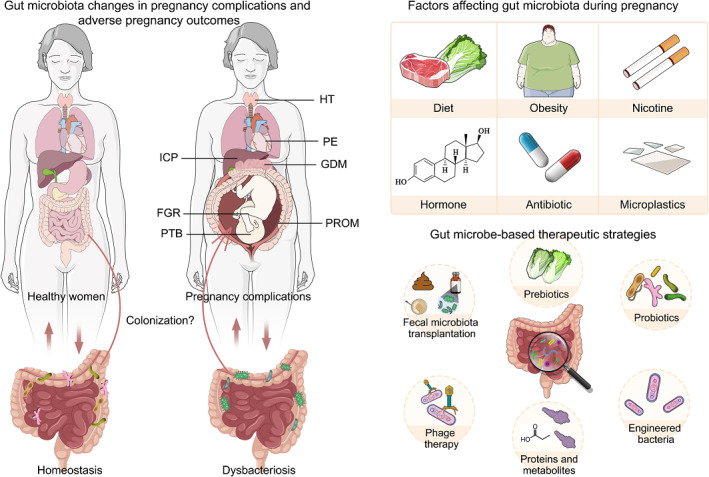

## INTRODUCTION

Pregnancy, a unique stage in a woman's life, involves significant physiological changes like hormonal, immune, and metabolic adaptations to support fetal development. Abnormalities in this process, including complications such as gestational diabetes mellitus (GDM) and pre‐eclampsia (PE), impact both the immediate and long‐term health of the mother and embryo. In 2023, the World Health Organization (WHO) reported that approximately 287,000 women globally succumbed to pregnancy‐related complications in 2020, averaging one death every 2 min [1]. This underscores the critical need for in‐depth research into the characteristics and mechanisms of these complications to protect women's and children's health.

The gut microbiota, exhibiting a complex symbiotic interaction with the host, is shaped by many factors including genetics, diet, hormone levels, delivery mode, and feeding method [2, 3]. Intriguingly, the human gut microbiota is likely to comprise a ratio closer to 1:1 in cell count compared to the human body [4, 5]. This ratio may vary among individuals due to factors such as body size and the amount of fecal material in the colon [4]. The gut microbiota, containing extensive genetic information and often regarded as the “second genome” and the largest endocrine organ in humans, is crucial for the host's immune, metabolic, endocrine, neural, and reproductive health [3, 6]. Extensive research has demonstrated that gut microbiota dysbiosis significantly contributes to various pathophysiological changes in the host and plays a pivotal role in the pathogenesis of numerous diseases, including cardiovascular, metabolic, and immune‐related conditions [3, 6].

During pregnancy, women's gut microbiota adapts to facilitate normal embryonic development and profoundly influences the structure and function of various host organs and tissues [7]. Consequently, dysbiosis of the gut microbiota inevitably impacts the health of both mother and fetus. Specifically, gut microbiota engages in complex interaction with reproductive hormones such as estrogen, significantly modulating the female reproductive endocrine system and crucially impacting women's reproductive health [8, 9]. A wealth of studies provide evidence associating gut microbiota dysbiosis during pregnancy with various complications, such as GDM, PE, fetal growth restriction (FGR), and preterm birth (PTB) [9, 10]. Nevertheless, much research in this area is still descriptive and insufficient. To uncover potential mechanisms, we offer specific experimental recommendations and suggest a path for commercialization or clinical translation.

In this review, from a pathophysiological perspective, we focus on the characteristic changes and etiological functions of the gut microbiota in pregnancy complications and their underlying mechanisms. Moreover, we discuss current insights on the possibility of existence of microbes in the uterus/placenta, which is critical for determining whether the gut microbiota impacts pregnancy complications directly or via certain metabolites, and for establishing the timeline of the origins of the infant gut microbiota. We also highlight the value of gut microbiota in the diagnosis and treatment of pregnancy complications and its great potential for clinical application through microbiota‐based interventions.

## IMMUNOLOGICAL AND HORMONAL CHANGES IN PHYSIOLOGICAL PROGRESSION OF NORMAL PREGNANCY

**Figure 1:**
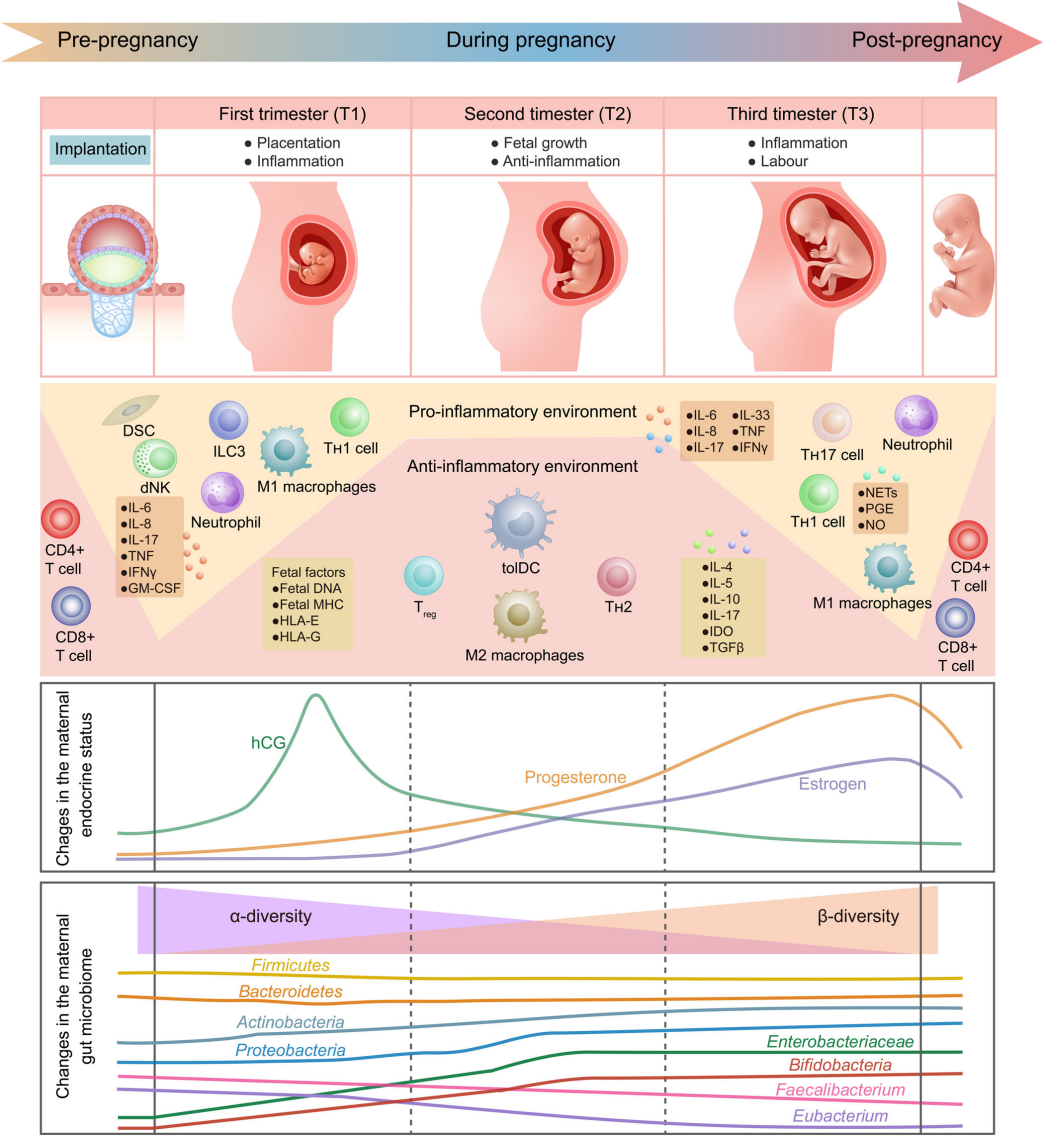
Evolution of immunological, hormonal, and gut microbiota profiles during physiological pregnancy. The changes in the immunological landscape during pregnancy are characterized by an early proinflammatory milieu in the first trimester (T1), conducive to postimplantation recovery. During the second trimester (T2), maternal–fetal immune tolerance develops within an anti‐inflammatory milieu, promoting fetal growth. In the third trimester (T3), the proinflammatory environment is re‐established, triggering the onset of labor. Concurrent hormonal changes are featured by a surge in human chorionic gonadotropin (hCG) levels early in gestation, which taper off gradually between Weeks 10 and 12, giving way to an escalating tide of progesterone and estrogen during T2. Microbiota dynamics are characterized by an initial reduction in α‐diversity and the amplification of Proteobacteria, Actinobacteria, and potentially pathogenic Enterobacteriaceae. Concurrently, the abundance of gut butyrate‐producing bacteria, such as *Faecalibacterium* and *Eubacterium*, declines. As T3 approaches, a significant bloom of *Bifidobacteria* readies the gut for impending childbirth. dNK cell, decidual natural killer cell; DSC, decidual stromal cell; GM‐CSF, granulocyte‐macrophage colony‐stimulating factor; hCG, human chorionic gonadotropin; HLA, human leukocyte antigen; IDO, indoleamine 2,3‐dioxygenase; IFNγ, interferon‐γ; ILC3, type 3 innate lymphoid cell; IL‐6, interleukin‐6; MHC, major histocompatibility complex; NETs, neutrophil extracellular traps; NO, nitric oxide; PGE, prostaglandin E; TGFβ, transforming growth factor β; tolDC, tolerogenic dendritic cells; TH1 cell, T helper 1 cell; TH2 cell, T helper 2 cell; TH17 cell, T helper 17 cell; TNF, tumor necrosis factor; Treg, regulatory T cell.

The embryo, inherently foreign and highly antigenic to the mother, requires a balanced immune response to maintain its immune privilege, crucial for a healthy pregnancy (Figure 1) [11]. Before pregnancy, inflammatory factors remain low. Implantation of the fertilized ovum triggers minor endometrial damage and human leukocyte antigen exposure on trophoblast cells, activating an innate immune‐driven proinflammatory process [12, 13, 14]. In early placental formation, the rapid surge of human chorionic gonadotropin (hCG) stimulates the proliferation of decidual natural killer cells in the decidua. These cells release cytokines and chemokines, vital for remodeling spiral arteries [13, 14, 15]. Activated group 3 innate lymphoid cells generate various inflammatory factors including interleukin‐17 (IL‐17), IL‐22, tumor necrosis factor (TNF), IL‐8, and granulocyte‐macrophage colony‐stimulating factor [14]. Thus, the implantation stage and the subsequent first trimester are characterized by a proinflammatory environment.

During the second trimester, immune tolerance peaks, alongside the production of anti‐inflammatory cells and factors, to shield the fetus from injury and rejection. The proliferation of regulatory T (Treg) cells (producing anti‐inflammatory cytokines such as IL‐10 and transforming growth factor‐β) is significantly enhanced, accompanied by the release of ovarian hormones such as estrogen and progesterone (anti‐inflammatory factors). The T‐cell populations at the placental–maternal interface shift from T helper 1 (Th1) (producing IL‐2, interferon‐gamma (IFNγ), and TNF) to Th2 (producing IL‐4, IL‐5, IL‐9, IL‐10, and IL‐13) and from Th17 (producing IL‐17) to Treg cells. These changes collectively buttress the embryo's immune privilege and are essential for maintaining placental–maternal tolerance [12, 16, 17].

Entering the third trimester, innate immune cells start a second proinflammatory process to trigger labor, releasing cytokines such as IL‐1, IL‐6, TNF, and matrix metalloproteinases from neutrophils and macrophages. Activated neutrophils increase the neutrophil extracellular trap (NET) formation, linked to PE [18]. Our previous study also reveals that NETs can promote the pathogenesis of another vascular inflammatory disease—abdominal aortic aneurysm [19]. After parturition, monocyte and neutrophil counts return to prepregnancy levels within 6 weeks, while T‐cell responses and CD4+/CD8+ proportions normalize over several months [20].

Dynamic hormonal changes are the central mechanisms sustaining pregnancy and preparing for childbirth. Following the fertilized ovum's implantation, the placental trophoblast begins to secrete hCG [21], which rises consistently, peaks between 10 and 12 weeks, and then gradually declines [22]. hCG contributes to blood vessels and placenta formation, contributes to fetal development, prevents uterine contractions, and concurrently stimulates progesterone and estrogen production [23]. Progesterone transforms stromal cells into decidual cells, maintaining endometrial stability and inhibiting premature uterine contractions [23]. Conversely, estrogen stimulates uterine vasodilation and blood flow increases to accommodate fetal growth and provokes uterine contractions in preparation for childbirth [24]. Additionally, the thyroid gland may become more active during pregnancy, potentially leading to hyperthyroidism (Figure 1) [25].

## CHARACTERISTIC CHANGES IN GUT MICROBIOTA DURING PHYSIOLOGICAL PREGNANCY

The composition and function of the maternal gut microbiota shift throughout pregnancy (Figure 1). A Finnish case–control study discovered that the first gut microbiome, primarily composed of Bacteroidetes and Firmicutes such as Clostridiales, shows similarities to that of nonpregnant women [26]. As pregnancy progresses from the first to the third trimester, the α‐diversity decreases and the β‐diversity increases, accompanied by an increase in Proteobacteria and Actinobacteria. Specifically, the Clostridiales order diminished, while the Enterobacteriaceae family increased [26]. Clostridiales is known for butyrate production, and its reduction weakens intestinal barrier function and anti‐inflammatory effects. Meanwhile, a rise in Enterobacteriaceae has been linked to the activation of inflammatory pathways [27, 28, 29]. This suggests that unfavorable microbiota changes in the later stages of pregnancy may be the onset of pregnancy complications. Coincidentally, complications such as GDM, PE, and intrahepatic cholestasis of pregnancy (ICP) often occur in the later stages of pregnancy [30, 31, 32]. Further causal research has found that in comparison to the first trimester, fecal microbiota of third trimester pregnant women can induce metabolic syndrome (MS) symptoms in germ‐free mice [26]. Another study showed increased levels of *Bifidobacterium* during the third trimester of both humans and mice, driven directly by progesterone [33]. This microbial shift seemingly prepares for neonate birth and initial breastfeeding, given *Bifidobacterium*'s documented potential for mother–infant transmission and the ability to metabolize human milk oligosaccharides, which is crucial to protect infants from infections and immune‐related diseases [34, 35]. These findings indicate that adaptive changes occur in the gut microbiota during pregnancy to support the increased metabolic demands and immune alterations, thereby maintaining normal pregnancy (Figure 1). However, other studies have reported conflicting results. One study found stable gut microbiota profiles during pregnancy with only a decrease in α‐diversity [36]. Another study reported no change in gut microbiota composition but potential shifts in metabolic activity [37].

Changes in α‐ and β‐diversity during physiological pregnancy vary across different longitudinal cohort studies. This inconsistency may be attributed to several factors, including age, obesity level, geographical location, short‐term dietary changes, small sample sizes, and participant characteristics [38]. To address these inconsistencies in gut microecology research, especially in cohort studies, the following should be considered: (1) conducting large‐scale, multicenter studies; (2) thoroughly collecting and recording factors influencing gut microbiota, demographics, and clinical data; (3) optimizing data analysis methods, using artificial intelligence methods like multivariable analysis and linear mixed models to explore diverse factors' effects and their interactions on gut microbiota, thereby identifying genuine microbiota changes linked to diseases; (4) employing techniques like CRISPR‐Cas, fecal microbiota transplantation (FMT), and washed microbiota transplantation (WMT); and (5) using antibiotic‐treated or germ‐free animal models to elucidate causal relationships between gut bacteria and diseases.

## INTERREGULATION OF GUT MICROBIOTA AND PREGNANCY‐RELATED HORMONES

During pregnancy, a subtle interplay forms between gut microbiota and hormones like estrogen and progesterone. As pregnancy progresses, gut microbiota changes directly or indirectly regulate the maternal hormonal environment via metabolites or enzyme activity to meet the demands of pregnancy. Understanding these gut microbiota–hormone interactions offers fresh insights and strategies for enhancing pregnancy health.

### Estrogen

Estrogen, particularly estradiol, is a key hormone in maintaining feminine traits and decreases with age. The gut microbiota significantly regulates estrogen levels and is influenced by estrogen [39]. The human gut metagenome is widely populated with genes encoding estrogen‐metabolizing enzymes, termed the “estrobolome” [40]. These enzymes (e.g., β‐glucuronidases, β‐glucosidases) deconjugate estrogen to promote intestinal reabsorption. Reduced intestinal flora diversity can lower β‐glucuronidase activity, inhibiting estrogen deconjugation and reabsorption in the intestinal tract, thereby decreasing the host's circulation and total estrogen burden [40]. Conversely, higher β‐glucuronidase‐producing bacteria (Table S1) may increase circulating estrogen [41]. *Klebsiella aerogenes TS2020*, known for its estradiol‐degrading properties, reduces serum estradiol levels by 3β‐hydroxysteroid dehydrogenase [42].

On the other hand, estrogen substantially affects gut microbiota, with estrogen receptor‐β (ERβ) in colonic epithelium crucial for microbiota balance. ERβ^−/−^ mice exhibit a reduced Bacteroidetes proportion (e.g., *Bacteroides thetaiotaomicron*) and an increase in Proteobacteria (e.g., *Escherichia coli*, *Desulfovibrio vulgaris*) compared to ERβ^+/+^ mice [43]. In mono‐associated rats, *B. thetaiotaomicron*, notable for acetate production, was found to enhance goblet cell differentiation, increase mucus‐related gene expression, and alter the balance of sialylated versus sulfated mucins [44]. Therefore, we speculate that *B. thetaiotaomicron* may help in estrogen‐mediated intestinal epithelial barrier repair [45].

Recent findings showed that gut microbiota dysbiosis induced by ERβ knockout triggered hyperactivity of the hypothalamic‐pituitary‐adrenal axis, which is associated with inflammatory bowel disease (IBD) and anxiety‐like behaviors [46]. Additionally, in male and ovariectomized female mice treated with 17β‐estradiol, there was a noticeable reduction in lipopolysaccharides (LPS)‐producing Proteobacteria and its subclasses, including γ‐Proteobacteria class, Enterobacteriaceae family, and the *Escherichia/Shigella* genus. For more representative LPS‐producing bacteria within the Proteobacteria phylum, see the literature [47] and Table S2. These findings suggest estrogen's potential role in decreasing LPS by gut microbiota, potentially leading to decreased intestinal permeability. This could further help in mitigating metabolic endotoxemia (ME) and low‐grade chronic inflammation, both of which are known to contribute to MS. Thus, the interplay between estrogen and gut bacteria plays a vital role in healthy pregnancy outcomes.

### Progesterone

Compared to estrogen, there are currently fewer studies regarding the connection between gut microbiota and progesterone. Recently, Kamimura et al. revealed that germ‐free mice have significantly lower fecal progesterone levels [48]. During pregnancy, elevated progesterone suppresses Th1 immune responses and inflammatory cytokines like IFNγ, while enhancing Th2 responses and anti‐inflammatory cytokines [49]. Nuriel‐Ohayon et al. discovered that nonpregnant female mice, with progesterone subcutaneously implanted for 21 days, exhibited substantial alterations in their gut microbiota, particularly an increase of *Bifidobacterium* [33]. *Bifidobacterium* offers health advantages by mitigating weight gain, improving insulin sensitivity and glucose tolerance, and reducing inflammatory substances such as IL‐6 from adipose tissue in pregnant animals on high‐fat diets [50, 51, 52]. Higher *Bifidobacterium* levels induced by progesterone may enhance pregnancy outcomes, as low levels are linked to PTB [53]. This is further supported by evidence showing that administering progesterone to women with a history of PTBs and a high risk of recurrence can decrease the likelihood of another PTB by 33% [54]. Another research conducted by Nuriel‐Ohayon et al. revealed that administering progesterone alters the gut microbiome in females, resulting in weight gain, an effect replicable in germ‐free mice through fecal transplant [55]. These findings illustrate a complex interaction between gut microbiota and progesterone.

### Androgens

In females, androgens are synthesized primarily in the adrenal cortex and ovaries [56]. Hannah Colldén et al. identified a decrease in the free form of dihydrotestosterone, in the lower intestinal tract of germ‐free mice, underlining the gut microbiome's significant impact on androgen metabolism [57]. Gut microbiome can regulate local and systemic androgen levels. For example, FMT from male mice to germ‐free mice can elevate serum testosterone levels [58]. Analogous to estrogens, androgens undergo conjugation in the liver and subsequently excreted into the intestine via bile. Certain gut bacteria, such as *E. coli*, have metagenomes encoding enzymes that deconjugate androgens, thereby increasing their absorption and circulation levels [57, 59]. Additionally, research indicates that prevalent intestinal microbiota, including *E. coli* and *Bacteroides* spp., are capable of synthesizing androgens from bile acids [60]. These findings imply that the gut microbiome has the capacity to regulate the exposure of intestinal epithelial cells to sex hormones through multiple pathways.

Excessive synthesis of androgens can induce diseases such as polycystic ovary syndrome (PCOS). In patients with PCOS, *Bacteroides vulgatus* are significantly higher. Transplanting gut microbes from patients with PCOS or from those colonized with *B. vulgatus* to recipient mice creates PCOS‐like conditions. These symptoms include a notable decrease in IL‐22 and glycodeoxycholic acid (GDCA) concentrations, significant impairment in ovarian activities, heightened insulin resistance, modifications in bile acid processing, and fertility issues [61].

On the other hand, the research conducted by Elin Org et al. showcased how androgens influence the microbiome. Their findings indicated a notable variation in the gut microbiota composition when comparing gonadectomized male rats to those that underwent sham surgeries. However, this difference was mitigated through dihydrotestosterone supplementation [62].

### Thyroid hormones

Triiodothyronine (T3) and thyroxine (T4) are key hormones in the regulation of metabolic processes and are integral to maintaining normal reproductive function in both sexes [8]. Thyroid hormones and gut microbiota exhibit a bidirectional relationship. In pregnant women, levothyroxine (LT4) treatment alters the abundance of various bacteria in thyroid peroxidase antibodies (TPOAb)‐positive subclinical hypothyroidism (SCH). This includes an enrichment of *Blautia*, *Streptococcus salivarius*, and *Bifidobacterium longum* in third trimester and a depletion of Bacteroidota, Bacteroidales, Bacteroidia, and *Prevotella* in second trimester, as well as *Agathobacter* in third trimester [63]. Xie et al., through Mendelian randomization analysis, determined that specific gut microbiota categories may play an etiological role in thyroid function at the genetic level and could potentially become effective biomarkers for the early diagnosis of thyroid‐related diseases. Among them, the phylum Actinobacteria exhibited a defensive role in preventing hypothyroidism, and the class Deltaproteobacteria demonstrated a protective effect against hyperthyroidism [64]. Our prior research has revealed gut microbiota dysbiosis in Graves' disease patients, and this phenotype and the increased T4 levels can be transferred by FMT, the underlying mechanism is achieved through modulating the differentiation of intestinal Treg and Th17 cell [65].

Abundant evidence supports the gut microbiota's mediating role in the metabolism of peripheral iodothyronines [66]. Intestinal microbes act as hydrolase executors and help maintain intestinal barrier integrity [67]. Studies have demonstrated that significant quantities of conjugated iodothyronines undergo hydrolysis within the fecal material [68]. A sort of obligate anaerobic bacteria possess glucuronidase activities and effectively hydrolyze conjugated T4 in the gut, facilitating hormone reabsorption into circulation and affecting the iodothyronine pool [69]. For example, even in small amounts like 3 × 10^7^/mL, *Peptococcus productus* can hydrolyze 50% of sulfated iodothyronines in 24 h [66, 70]. Some researchers even consider the gut microbiota to be a component of the intestinal barrier. In germ‐free mice, microbiota deficiencies reduce intestinal surface area, potentially disrupting the oral T4 reabsorption process through the enterohepatic circulation [66, 71]. The effect of microbiota on thyroid hormone balance is a fascinating research topic. Studies show that thyroid disorders during pregnancy, like hypothyroidism or hyperthyroidism, can lead to a higher risk of miscarriage and PTB. This indicates that gut microbiota might affect reproductive health by changing thyroid function.

## NONHORMONAL FACTORS AFFECTING GUT MICROBIOTA DURING PREGNANCY

### Diet

Diet significantly impacts gut microbiota [72]. Pregnant women generally exhibit higher diet quality scores than those trying to conceive or in their reproductive years, often due to increased awareness of the importance of nutrition. As a result, they often increase their intake of nutrient‐rich foods, prioritizing key components such as protein, sugar, fat, fiber, and various vitamins [73].

A fiber‐rich diet during pregnancy enhances gut microbiota richness and diversity, reducing the levels of *Collinsella* and *Holdemania*, while promoting the growth of bacteria known for producing short‐chain fatty acids (SCFAs), including *Roseburia* and Lachnospiraceae. This shift elevates SCFA levels, promoting gut mucosal health and mitigating inflammation [74, 75]. In contrast, the Western diet triggers a generational depletion in “ancestral” gut microbial diversity and dysbiosis, contributing to adverse maternal and infant health outcomes [76]. High carbohydrate intake during pregnancy is positively correlated with an abundance of Proteobacteria and Bacteroides, while being negatively correlated with Firmicutes (e.g., the Ruminococcaceae family and *Ruminococcus* genus) and *Roseburia*. Conversely, lower carbohydrate intake is linked with an increased *Lachnospira* [77].

The second dietary change during pregnancy is an increases intake of protein‐rich beef [78]. High total protein intake, particularly from animal proteins, tends to reduce Actinobacteria and Proteobacteria and increase *methanogens*, important for hydrogen removal and nutrient energy yield [74, 79]. High sugar diets reduce the synthesis of the microbial metabolite indole, contrary to protein‐rich diets that increase it. Indole is beneficial for glucose metabolism and lowering blood lipid levels [80, 81]. Consequently, the Mediterranean diet, known for naturally enhancing indole concentrations, acts as a potential protective factor in metabolic regulation [82]. However, it should be noted that many fruits contain high levels of sugar, such as juices, apples, grapes, and watermelon, as well as peas, asparagus, and zucchini. Additionally, the fructose supplementation during gestation significantly reduces *Lactobacillus* and *Bacteroides*, thereby impairing gut barrier function in rat offspring [83]. Therefore, consuming a moderate amount of foods with lower fructose content, such as bananas, strawberries, blueberries, avocados, lettuce, and green beans, is advisable for pregnant women.

According to Public Health Institute standards, approximately 35% of pregnant women consume excessive amounts of fat, compared to 23% of nonpregnant women [78]. A high‐fat diet increases intestinal *Akkermansia*, known for its mucin‐degrading properties, while reducing butyrate‐producing genera such as *Lachnospira* and *Ruminococcus*. It also enhances the abundance of *Collinsella* spp. in the colon, disrupts the gut barrier integrity, and stimulates the production of IL‐17A, which potentially influences placental angiogenesis and fetal gut development [84, 85].

Additionally, many pregnant women frequently take vitamins and folic acid [73]. A diet rich in vitamin D has been linked to a higher abundance of Actinobacteria and Proteobacteria, which include many low pathogenicity species and *Staphylococcus* [79]. Monounsaturated fatty acids, cholesterol, and vitamin A are also correlated with an increase in Proteobacteria that contain pathogens and exhibit proinflammatory properties [79]. However, higher vitamin E intakes were associated with lower Proteobacteria and *Sutterella* levels, the latter being more prevalent in infants with autism and gastrointestinal disorders [86], indicating potential health benefits of vitamin E [79]. Animal studies reported that pregnancy diets rich in vitamins or imbalanced in folic acid and choline can lead to gut microbiome dysbiosis, potentially increasing obesity risk in offspring [87]. Therefore, not all types of vitamins uniformly benefit gut microbiota health, highlighting the need for controlled intake and mindful selection of vitamins and folate during pregnancy.

Another change in the dietary behavior of pregnant women is the increased consumption of milk and dairy products [78]. The impact of maternal milk intake on the mother's own gut microbiota has not been extensively reported, but research suggests a connection between consuming cow's milk during breastfeeding and a reduced incidence of food allergies in their children [88]. The influence of fermented dairy products like yogurt will be discussed later.

These findings highlight the substantial impact of maternal diet on gut microbiota, with potential lifelong effects on mother and child health. Future gut microbiota research faces the challenge of reducing pregnancy complications through fiber‐rich diets and personalized nutrition, aiming to restore the “ancestral” gut microbiome characteristic of preindustrialized, plant‐rich diets. Such a diet nurtured a gut microbiome proficient in processing microbiota‐accessible carbohydrates, promoting a human genome that synergizes well with these fibrolytic microbes and their metabolites. This synergy could decrease chronic inflammation and obesity cases, lowering the risk of noncommunicable chronic diseases [89].

### Obesity

Obesity, the most prevalent metabolic disorder during pregnancy, is closely associated with adverse outcomes such as placental hypoxia [90]. Obese pregnant women present an increased Firmicutes‐to‐Bacteroidetes ratio and a substantial reduction in gut microbiota diversity in late gestation [91]. Maternal diet‐induced obesity results in lower intestinal levels of SCFA‐producing Lachnospiraceae, butyric acid, and SCFA receptors messenger RNA [90]. A significant decline in beneficial bacteria such as *Bifidobacteria* and *Lactobacilli* and an increase in potential harmful bacteria such as *E. coli*, Enterobacteriaceae, and *Staphylococcus aureus* are observed in the gut of obese pregnant women [76, 92]. Moreover, alterations in the gut microbiota of obese pregnant women have substantial implications for their offspring. Infants born to overweight and obese mothers are more susceptible to obesity, possibly due to transmission of specific bacteria and metabolites. Fecal samples from these infants showed a markedly reduced abundance of *Ruminococcus*, *Blautia*, and *Eubacterium* in the phylum Firmicutes, whereas *Oscillibacter* and *Parabacteroides* are more prevalent. This shift may elevate the risk of metabolic diseases in offspring [93, 94, 95]. In summary, obesity is intimately tied to an imbalance of gut microbiota, metabolic disorders, and systemic inflammation in pregnant women.

### Nicotine exposure

Nicotine, the primary hazardous component of tobacco, enters the body through inhalation, dermal absorption, and gastrointestinal tract. Nicotine intake during pregnancy is considered a major risk factor for complications, potentially due to its association with gut microbiota dysbiosis [96]. Nicotine significantly decreases beneficial intestinal bacteria (e.g., *Bifidobacterium* and *Lactobacillus*), while increasing pathogenic bacteria (e.g., *E. coli* and *Klebsiella pneumoniae*) in pregnant rats [97]. In the samples of colon and plasma from healthy pregnant women, levels of propionic acid, butyric acid, and caprylic acid are positively correlated, but nicotine exposure disrupts these relationships. This suggests that nicotine may interfere with the production and absorption of intestinal SCFAs [97]. Another study indicated that prenatal nicotine exposure induces an increase in Actinobacteria in pregnant rats, potentially causing an increase in Firmicutes and a decrease in Verrucomicrobia. Furthermore, nicotine downregulates several genes in the proximal colon microbiota of pregnant women, which are typically upregulated during pregnancy, such as free fatty acid receptor 2 (*FFAR2*), the acetate and propionate transporter, and tryptophan hydroxylase 2 (*TPH2*), the rate‐limiting enzyme for the production of 5‐hydroxytryptamine [98]. Cumulatively, these findings confirm that nicotine exposure can markedly modulate the gut microbiota in pregnant women, potentially jeopardizing fetal development by affecting the gut‐derived metabolite bioactive molecules.

### Antibiotic use

Antibiotics, especially broad‐spectrum types, can significantly and persistently alter the gut microbiota, reducing its taxonomic abundance and diversity. Such disruption can upset the delicate equilibrium between beneficial and harmful bacteria, promoting the proliferation of antibiotic‐resistant bacteria and health issues [99, 100]. Li et al. reported that perinatal antibiotic exposure disrupts the mother‐to‐offspring commensal microbiota transmission during the pivotal period of initial microbial colonization, allowing environment‐derived bacteria, including hospital‐associated strains, to colonize the neonatal intestine [101]. Perinatal antimicrobial prophylaxis (IAP) is the most common route of perinatal and neonatal antibiotic exposure [102]. Many studies underscored the effect of IAP on gut microbiota development, including an increase in Enterobacteriaceae and a decrease in anaerobic bacteria such as *Bifidobacterium* and *Bacteroides* [103]. IAP also negatively hampers the normal establishment of the *Bifidobacterial* community in newborns [104]. Numerous investigations have identified a dose‐dependent correlation between maternal antibiotic use and increased risks of eczema, food allergies, asthma, and obesity in offspring [105]. Although life‐saving, antibiotics may engender complex influences on maternal and fetal gut microbiota. Prescribing antibiotics during pregnancy requires careful deliberation, balancing benefits and risks to minimize potential harm.

### Microplastics (MPs)

MPs, including particles <5 mm and nanosized plastics <1 μm, are ubiquitous in the global biosphere, contaminating everything from the air to our food. This widespread pollution is of growing concern for the health of infants and pregnant women [106]. MPs have been discovered in human placenta, meconium, and infant stool [107, 108], yet tracking their origins is challenging. Inhalation, placental transfer, breastfeeding and ingestion, and dermal absorption all could potentially serve as exposure pathways for child‐related MPs [109].

A study presents the initial evidence linking MPs to microbiota in human placentas and meconium. It found 16 varieties of MPs across all samples and an inverse relationship between MPs (specifically polystyrene and polyethylene particles sized 50–100 μm) and several genera in the placental and meconium microbiota, suggesting MPs influence microbiota composition [110]. Another study showed that maternal exposure to polystyrene MPs during gestation and lactation caused metabolic and gut microbiota imbalances in maternal mice, with intergenerational effects like persistent liver lipid metabolism dysregulation in their offspring [111]. Additionally, chronic exposure to MPs exacerbated liver damage elicited by cyclophosphamide, significantly linked to colon impairments including increased intestinal permeability, mild inflammation, and reduced antioxidant function, as well as gut microbiota disturbances. These findings were further validated through FMT experiments [112]. To date, most research on MPs focuses on marine environments, with limited studies on MPs and gut microbiota, hindering long‐term tracking of MP concentrations. Furthermore, there is also a lack of longitudinal cohort studies on the relationship between MPs and maternal and child health.

To mitigate the impact of MPs on maternal and child health, we recommend food manufacturers optimize their design and materials to minimize MP generation and contamination and verify product safety. Interdisciplinary research is essential to fully understand the effects of early‐life MP exposure on gut microbiota and health. Mother–infant cohort studies should include microplastic exposure considerations, collecting relevant samples and data. Families can take measures to reduce children's exposure to MPs, such as avoiding food contact with plastic, choosing safer personal care products and building materials, and selecting plastic‐free or low‐plastic options for baby products and toys.

## DYSBIOSIS AND ITS ETIOLOGICAL ROLES OF GUT MICROBIOTA IN PREGNANCY COMPLICATIONS

Currently, there is still controversy regarding the relationship between pregnancy complications and adverse pregnancy outcomes. One perspective defines an adverse pregnancy outcome as any event that diminishes the possibility of delivering a healthy baby. This includes conditions like PTB, FGR, fetal anomalies, stillbirth, intrauterine fetal death, and infants small for gestational age. These outcomes have been associated with specific pregnancy complications, such as PE and GDM [113, 114]. However, there are also some viewpoints that classify PTB, FGR, and other obstetric abnormalities as part of pregnancy complications [115, 116]. In this context, we categorize PTB and FGR as adverse pregnancy outcomes.

### GDM

GDM is a transient hyperglycemia during pregnancy [30]. Its prevalence ranges between 5.4% and 14%, and it is considered a prediabetic state [117]. Compared to healthy pregnant women at the same gestational stage, the gut microbiota of GDM women is markedly disturbed, akin to the typical composition in nonpregnant type 2 diabetes patients [30, 118, 119]. For instance, *Bacteroides*, *Parabacteroides distasonis*, and *Klebsiella variicola* are significantly enriched in the guts of women with GDM. Conversely, *Alistipes* spp., *Methanobrevibacter smithii*, *Bifidobacterium* spp., and *Eubacterium* spp. are massively enriched in the intestines of normoglycemic pregnant women [120]. Recently, Sun et al. clarified the correlations between 10 specific microbial species and GDM. For example, *Alistipes putredinis* and *Bifidobacterium dentium* are negatively correlated with GDM, while *E. coli*, a member of the Enterobacteriaceae family, is positively correlated with GDM, glycemic indicators (fasting glucose, glycated hemoglobin), and weight parameters (body mass index, gestational weight gain). Remarkably, a GDM risk score based on these 10 bacteria is consistently higher in GDM patients than that in controls throughout pregnancy [121]. Dietary fiber‐rich foods affect the impact of these bacteria on glycemic indices, with metabolic pathways involved in fiber degradation strongly negatively correlating with fasting glucose levels [121]. Despite variations in the microbial taxa associated with GDM across studies, there is generally an increase in Enterobacteriaceae, *Desulfovibrio*, *P. distasonis*, Ruminococcaceae, *Prevotella*, and *Collinsella* and a decrease in *Alistipes* spp., *Faecalibacterium*, and *Bifidobacterium* [121, 122]. These studies collectively demonstrate a strong link between gut microbiota changes and GDM. Endotoxin‐producing opportunistic pathogens such as Enterobacteriaceae are enriched in GDM patients [123], which correlates positively with GDM in Sun et al.'s study. Conversely, keystone species responsible for starch degradation and SCFA production, such as *Alistipes* and *Bifidobacterium*, which have shown beneficial effects in preventing inflammation and insulin resistance [124, 125], are depleted in patients with GDM, as shown by their negative correlation with GDM in Sun et al.'s study. Therefore, these three categories of gut bacteria are likely involved in the development of GDM.

**Figure 2:**
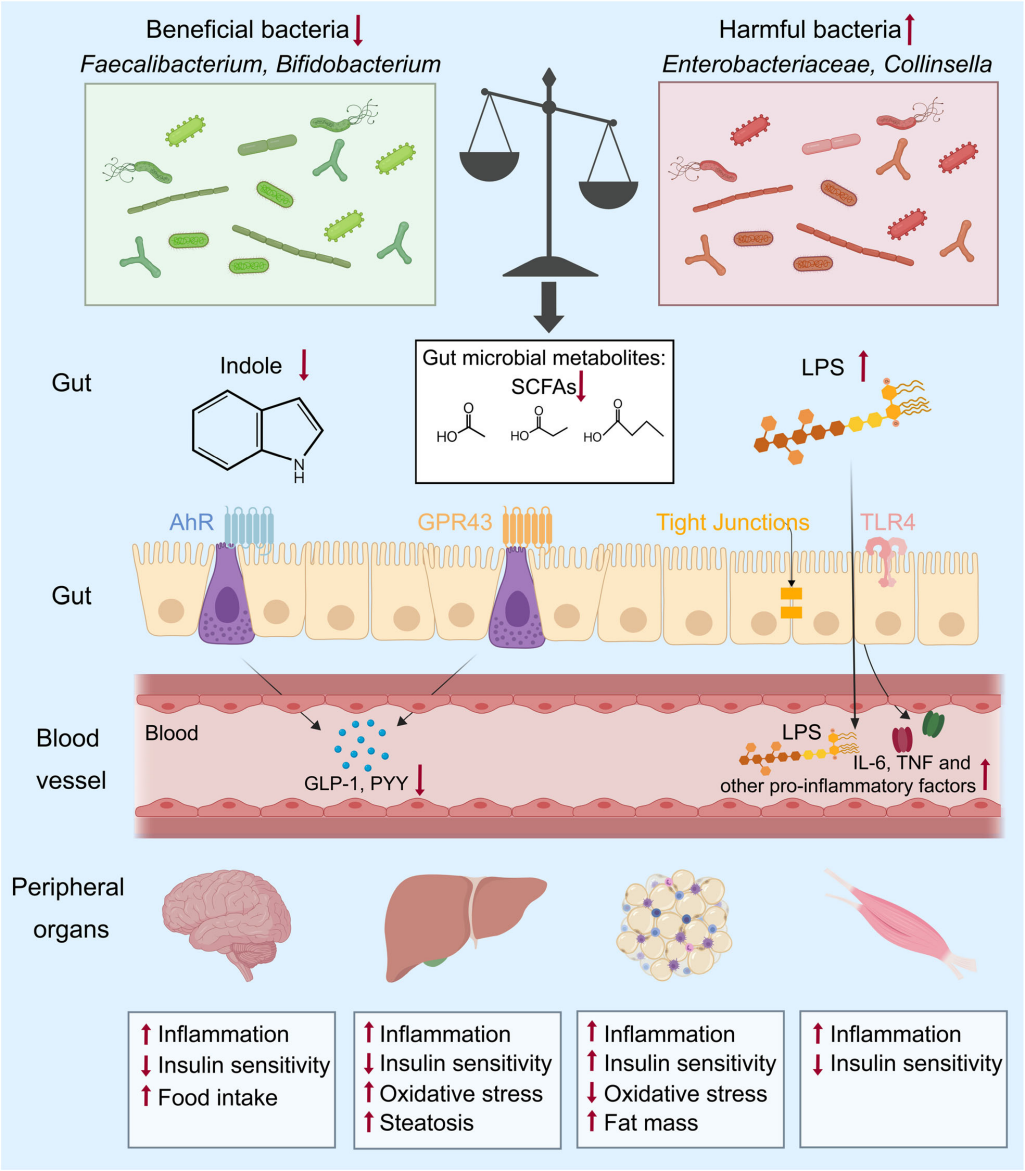
Conceptual depiction of mechanisms for gestational diabetes mellitus (GDM). Maternal gut dysbiosis worsens GDM outcomes. Short‐chain fatty acids (SCFAs) mainly produced by gut microbes *Faecalibacterium* and *Bifidobacterium* bind to G protein‐coupled receptor 41 (GPR41) and GPR43, facilitating the release of glucagon‐like peptide‐1 (GLP‐1) and peptide tyrosine tyrosine (PYY) from enteroendocrine L‐cells. Indoles, the metabolic products of aromatic amino acids (AAAs), stimulate GLP‐1 release via the aryl hydrocarbon receptor (AhR) pathway. Lipopolysaccharides (LPS) synthesized by gut Enterobacteriaceae increase the levels of certain proinflammatory factors in the gut through Toll‐like receptor‐4 (TLR4). During pregnancy, gut dysbiosis leads to a reduced SCFA levels, diminishing GLP‐1 and PYY influx into circulation and concurrently escalating LPS levels, thereby promoting excessive LPS and proinflammatory factors entry into the bloodstream. These shifts further contribute to enhanced inflammation and decreased insulin sensitivity in extraintestinal tissues and organs including brain, muscles, liver, and adipose tissue, ultimately leading to the onset of GDM.

**Figure 3:**
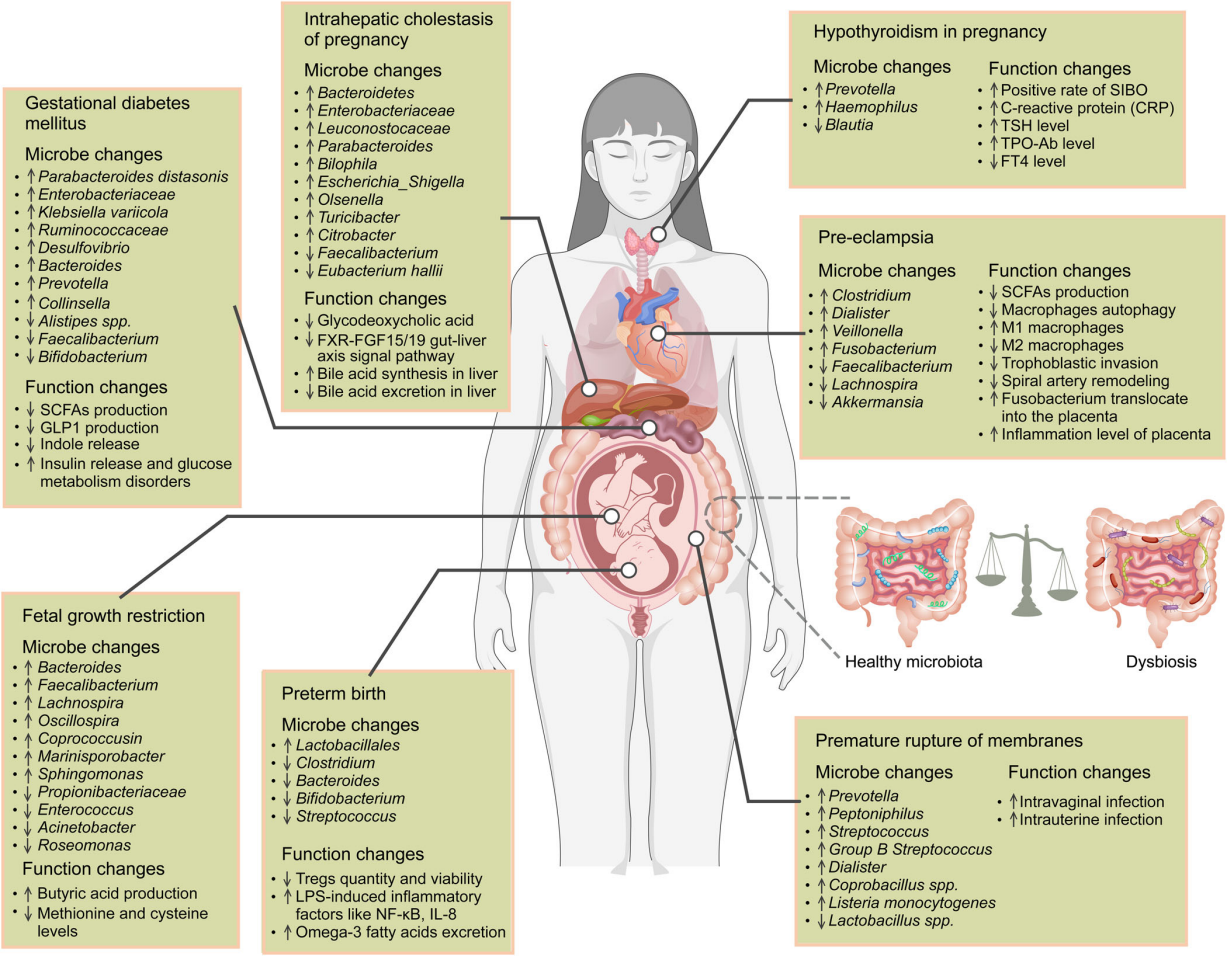
Enumeration of intestinal microbes and associated functional changes in pregnancy complications. This figure summarizes the findings from metagenomic and 16S ribosomal RNA sequencing studies, outlining the selected microbes and associated functional features alongside their tendencies in pregnancy complications. Despite discrepancies across studies, the curated list shows common patterns presented in different research. While not exhaustive of all microbes or functional changes, it represents those most frequently observed in association with pregnancy complications. CRP, C‐reactive protein; FGF, fibroblast growth factor; FT4, free thyroxine; FXR, farnesoid X receptor; IL‐8, interleukin 8; NF‐κB, nuclear factor‐κB; SIBO, small intestinal bacterial overgrowth; TPO‐Ab, thyroid peroxidase antibody; TSH, thyroid‐stimulating hormone. ↑ indicates higher levels in pregnancy complications compared with control; ↓ indicates lower abundance in pregnancy complications compared with control.

A decline in SCFAs, key gut microbiota metabolites including acetate, butyrate, and propionate, correlates with glucose metabolism disturbances. SCFAs can bind to G protein‐coupled receptor 41 (GPR41) and GPR43, inducing the release of peptide tyrosine tyrosine and glucagon‐like peptide‐1 (GLP‐1) from enteroendocrine cells, thereby regulating insulin release and glucose metabolism [126]. Additionally, bacteria that degrade aromatic amino acids (AAAs) are significantly reduced in patients with GDM. Indoles, intestinal metabolites of AAAs, can stimulate GLP‐1 release via the aryl hydrocarbon receptor pathway [127, 128]. Multiple studies have reported various beneficial effects of indole and its derivatives, such as protecting mucosal intestinal epithelial cell function, its anti‐inflammatory properties, improving insulin resistance, inhibiting LPS translocation, and reversing metabolic dysfunction [129, 130]. However, inconsistent research findings exist. For instance, higher serum and fecal indole concentrations have been observed in women with GDM compared to non‐GDM women, with a positive correlation between indole and GDM risk [131, 132]. Notably, the GDM phenotype can be effectively transmitted through FMT (Figures 2 and 3) [133].

### PE

PE is a pregnancy‐specific syndrome, defined by new‐onset hypertension after 20 weeks of gestation and at least one accompanying complication, such as proteinuria, maternal organ dysfunction (e.g., renal insufficiency, liver impairment, neurological or hematological disorders), or uteroplacental dysfunction (e.g., FGR) [31]. Arguably, PE is one of the most serious complications of pregnancy and is a leading cause of perinatal mortality worldwide. Currently, there is no effective treatment for PE other than the termination of pregnancy [31, 134].

**Figure 4:**
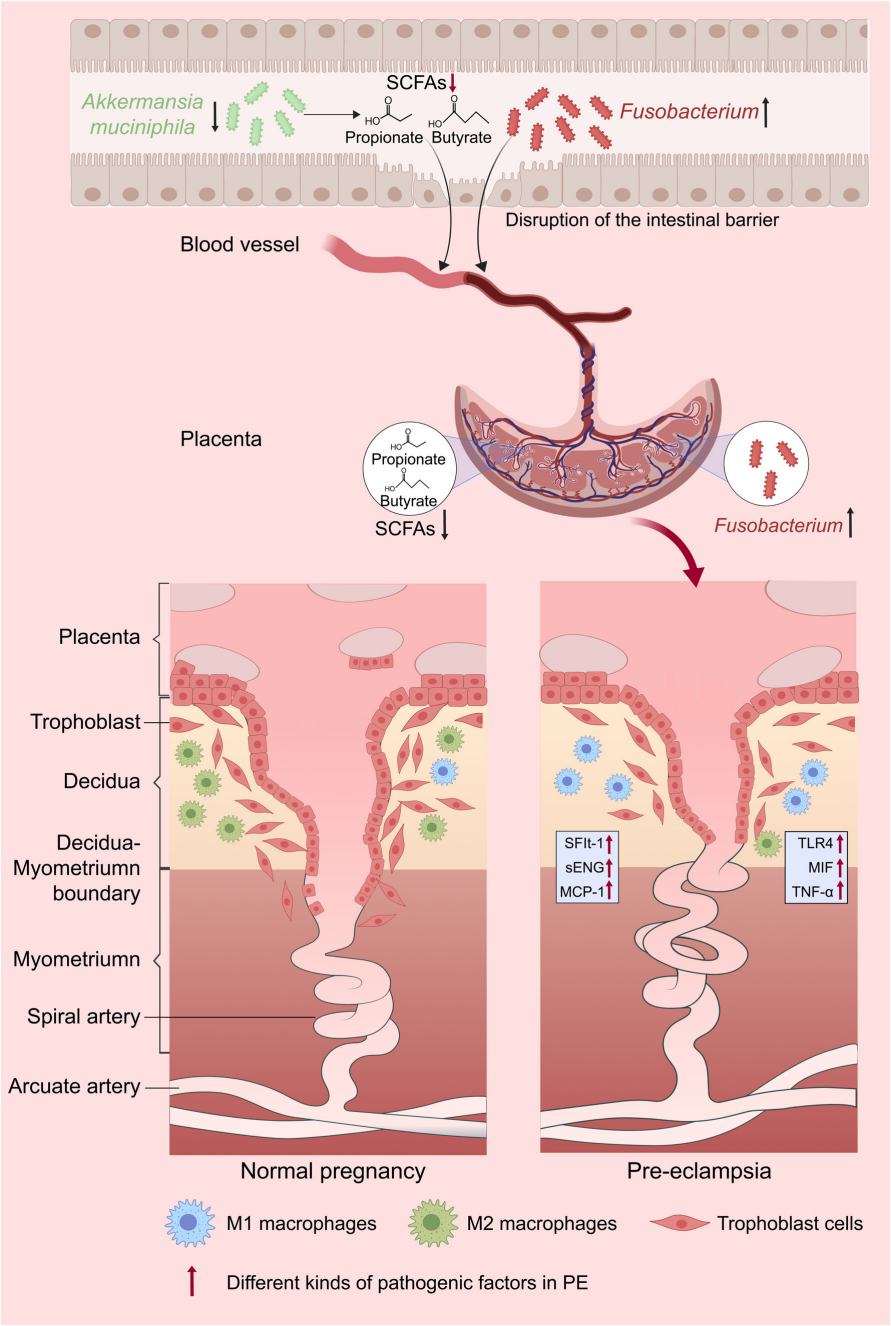
Schematic representation of mechanisms underpinning pre‐eclampsia (PE). Maternal gut dysbiosis drives the progression of PE. In PE patients, gut leakage coincides with a significant decline in *Akkermansia*, a key contributor to SCFA production. This reduction leads to a paucity of circulating SCFAs available to the placenta, triggering a series of events: hypertension ensues, autophagy in placental macrophages is inhibited, the transformation from M2 to M1 macrophage phenotype is enhanced, and trophoblast invasion is impaired. These events subsequently contribute to abnormalities in embryonic and placental development, as well as abnormal remodeling of spiral arteries. Concurrently, a pronounced increase in *Fusobacterium*, together with gut leakage in PE patients, facilitates its translocation into the placenta, thereby exacerbating placental inflammation and causing abnormal growth. Collectively, these perturbations orchestrate the onset of PE. MCP‐1, monocyte chemoattractant protein‐1; MIF, macrophage migration inhibitory factor; sENG, soluble endoglin; sFlt‐1, soluble fms‐like tyrosine kinase‐1.

Studies have highlighted notable gut microbiota differences in PE patients compared to healthy pregnant women. Research in China identified increased *Bulleidia moorei* and *Clostridium perfringens* and decreased propionate‐producing *Coprococcus catus* in PE patients [39]. A recent large‐scale Mendelian randomization study confirmed the causal link between *Bifidobacterium* and reduced risk of PE, providing a clue for further investigation into the protective effects of probiotics against PE [135]. In a study published in *Gut*, a 16S sequencing analysis of fecal microbiota from 67 PE patients and 85 healthy pregnant women revealed lower α‐diversity and significant dysbiosis in the gut microbiota of PE patients [136]. In detail, pathogenic genera such as *Clostridium* (exc. *Clostridium butyricum*, a beneficial member), *Dialister*, *Veillonella*, and *Fusobacterium* were significantly enriched, while probiotic genera like *Lachnospira*, *Akkermansia*, and *Faecalibacterium* were diminished. These microbial alterations correlated with clinical indicators of PE, such as blood pressure and biomarkers of liver and kidney function. Gut microbiota from PE patients could markedly raise blood pressure in prepregnant mice. After pregnancy, these mice exhibited further elevated blood pressure, accompanied by proteinuria, embryonic resorption or disintegration, reduced fetal–placental weight, and placental inflammation. These findings illuminate the critical causal role of gut microbiota dysbiosis in PE pathogenesis. Mechanistically, small intestinal T‐lymphocyte disruption and intestinal leakage allowed bacteria such as *Fusobacterium* to translocate into the placenta, increasing inflammation and impairing growth [136]. Our study likewise found gut microbiota dysbiosis in PE patients, with reduced SCFA‐producing bacteria and lower placental propionic and butyric acids. *Akkermansia muciniphila*, propionic acid, or butyric acid could ameliorate hypertension, embryonic/placental dysgenesis, and poor spiral artery remodeling in pre‐eclamptic rats [137]. Mechanistically, these agents enhanced autophagy in placental macrophages and inhibited their M1 phenotypic conversion. Propionic acid was found to promote macrophage autophagy, M2 polarization, and trophoblast invasion via activation of AMPK‐mTOR, GPR43‐STAT1, and GPR41‐AKT signaling pathways. However, the cycle threshold values of bacteria in the peripheral blood and placenta of pregnant women detected by quantitative polymerase chain reaction exceeded the sensitivity of the PCR instrument, providing no evidence of intestinal bacterial translocation to the placenta via the bloodstream. Therefore, we prefer that gut bacteria regulate PE occurrence through their metabolites, SCFAs, rather than the bacteria themselves (Figures 3 and 4) [137]. The translocation of gut bacteria and their presence in the placenta will be discussed later.

Although direct evidence linking gut microbiota‐derived indole and its derivatives to PE is currently lacking, emerging research suggests indole's positive effects on immune regulation and vascular function. For example, indole‐3‐aldehyde, produced by *Lactobacillus reuteri*, showcases its beneficial properties by preventing vascular smooth muscle cells phenotypic switch, minimizes macrophage infiltration, and reduces the release of inflammatory cytokines, collectively postpone aortic dissection progression [138]. Meanwhile, *L. reuteri*, *Akkermansia*, and the *Clostiridum* genus are key in producing indole‐3‐propionic acid [82], which combats oxidative stress, lipid peroxidation, and inflammation at the cellular level. Intriguingly, its synthesis is observed to decrease in individuals with various histological classifications of atherosclerosis [82]. These findings underscore the potential of indole as a promising intervention strategy for the prophylaxis of vascular diseases. Given the previously validated role of SCFAs generated by *Akkermansia* in PE [137], exploring indole metabolites produced by *Akkermansia* and other bacteria is also pertinent in PE research.

### ICP

ICP is the most common pregnancy‐specific liver disease, occurring mainly in the second and third trimesters, characterized by maternal pruritus and hepatic dysfunction, including elevated serum bile acids and transaminases [32]. ICP increases the risk of adverse perinatal outcomes, such as FGR, spontaneous PTB (SPTB), low Apgar scores, and even intrauterine death [139]. The etiology and pathophysiology of ICP remain unclear.

Many studies have shown significant abnormalities in gut microbiota in patients with ICP compared to healthy individuals [140]. For instance, a high relative abundance of Bacteroidetes and a pronounced deficiency of SCFA‐producing bacteria (e.g., *Faecalibacterium*, *Blautia*, and *Eubacterium hallii*) have been noted. Enterobacteriaceae and Leuconostocaceae, along with rare bacterial species (e.g., *Blautia* and *Citrobacter*), were found to be elevated in ICP patients [141]. *Escherichia_Shigella*, *Turicibacter*, and *Olsenella* were significantly enriched in severe ICP cases [140].

Bacteria enriched in ICP patients are closely associated with bile acid metabolism, inflammation, and cirrhosis (e.g., *Bilophila* and *Parabacteroides*) [142]. The bile salt hydrolase (BSH) activity of some gut bacteria influences bile acid dihydroxylation and deconjugation, regulating the bile acid pool [143, 144]. The farnesoid X receptor (FXR) is expressed in the ileum and liver and fulfill a critical role in regulating bile acid homeostasis [139]. Recent studies show an enrichment of *Bacteroides fragilis* in the intestines of ICP patients. *B. fragilis* can directly mediate the deconjugation of GDCA via its BSH activity, thus inhibiting the FXR‐FGF15/19 enterohepatic signaling pathway, promoting hepatic bile acid synthesis and exacerbating ICP. Therefore, modulating the gut microbiota–bile acid–FXR axis may be beneficial for ICP treatment [139]. However, some studies demonstrated a reduction in BSH‐active bacteria in the intestine, leading to an increase in conjugated bile acids and an inhibition of intestinal FXR pathways, resulting in elevated hepatic bile acid synthesis [145]. This discrepancy may be attributed to different BSH‐active bacteria targeting different types of conjugated bile acids (Figure 3).

### Hypothyroidism in pregnancy

Hypothyroidism in pregnancy is characterized as a systemic hypometabolic syndrome, typically due to hypothyroxinemia or thyroid hormone resistance, presenting with an incidence of approximately 0.5%. The etiology of this disorder remains elusive [146]. Despite research exploring the gut microbiota composition in individuals with thyroid dysfunction [147, 148], attention has only recently turned to hypothyroidism in pregnancy. A study of pregnant women with SCH indicated a correlation between small intestinal bacterial overgrowth (SIBO) and SCH. Compared to healthy controls, the prevalence of SIBO is elevated in SCH patients, who also exhibit higher levels of C‐reactive protein (CRP) and an increased incidence of bloating and constipation. Moreover, SIBO was positively associated with thyroid‐stimulating hormone and TPOAb levels and inversely correlated with free thyroxine (FT4) levels [149]. In 2022, Wu et al. disclosed that *Faecalibacterium*, *Bacteroides*, *Prevotella 9*, *Bifidobacterium*, *Subdoligranulum*, *Lachnospira*, and *Megamonas* were the dominant bacterial genera in pregnant women with SCH. Levothyroxine (LT4) treatment influenced the gut microbiota composition, implying that the gut microbiota could be a novel therapeutic target [150]. Additional studies have revealed distinct gut microbiota in hypothyroid pregnant women, with higher *Prevotella* and *Haemophilus* and lower *Blautia* compared to healthy controls (Figure 3) [151]. Clearly, research on the gut microbiota and thyroid dysfunction during pregnancy is still in a descriptive phase compared to other diseases.

Subsequent research should primarily focus on the following areas: (1) directly measuring the microbial composition in feces of pregnant patients with Hashimoto's thyroiditis or Graves' disease on propylthiouracil (PTU) or TPOAb‐positive SCH patients on LT4, alongside checking plasma PTU or LT4 levels. This will clarify thyroid hormones' impact on gut microbiota; (2) conducting experiments to elucidate causal relationships between gut microbiota and thyroid dysfunction during pregnancy; (3) performing preclinical studies to uncover the underlying mechanisms of characteristic bacteria involved in pregnancy‐related thyroid dysfunctions. Based on these fundamental studies, further clinical trials can be conducted to test probiotics, such as *B. longum*, known to improve thyroid function [152], and other promising probiotics found in preclinical studies, to evaluate their effectiveness in pregnant women with thyroid disorders for clinical application.

### Premature rupture of membranes (PROM)

PROM accounts for nearly one‐third of SPTBs and is a high‐risk factor for neonatal sepsis and mortality [153]. Bacterial infections and the ensuing inflammatory responses are key contributors to PROM [154]. Traditionally, the uterine cavity is viewed as a sterile environment, which has been challenged by the detection of bacterial DNA through PCR methods [155, 156]. Ascending infections from the vagina are considered the primary cause of PROM, but gut bacteria migrating to the vagina and amniotic fluid suggest they might also cause intrauterine infections linked to PROM [157, 158]. *Listeria monocytogenes* can traverse the intestinal mucosal barrier and infect the placenta via hematogenous transmission, which lends further support to this notion [159]. Research has suggested that the gut microbiota of pregnant women might relocate to the vagina, altering vaginal flora. Oral administration of *Lactobacillus* can alter vaginal flora and inflammatory response, underscoring the potential relationship between gut and vaginal microbiota [160]. Generally, compared to gut microbiota, a lower abundance and diversity of vaginal flora in nonpregnant women is considered beneficial in maintaining healthy and stable pregnancy [161]. However, women with PROM show complex, abnormal vaginal microbiota with increased diversity and pathogens. These bacteria can ascend, colonize placental tissue, cause chorioamnionitis, and trigger PROM [162, 163]. Overall, these findings suggest that gut microbiota may directly or indirectly influence the intrauterine environment, playing a key role in PROM and offering avenues for new preventive and therapeutic approaches (Figure 3).

### Maternal dyslipidemia and hypertension during pregnancy

Several studies have highlighted the involvement of gut microbiota in the development of maternal dyslipidemia and hypertension during pregnancy. Many pregnant women develop gestational hypertension (GH) before PE occurs. In 2023, evidence from Mendelian randomization revealed a protective effect of the probiotics *Bifidobacterium* and *Intestinibacter* on GH [164]. A nested case–control study observed gut microbiota dysbiosis in pregnancy‐induced hypertension (PIH) patients, including a reduction in beneficial bacteria like *A. putredinis* and *A. muciniphila* from early pregnancy. Bacterial pathway analyses indicate that this dysbiosis may contribute to the development of PIH by affecting the metabolism of vitamin K2, sphingolipids, fatty acids, and glycine [165].

Regarding lipid metabolism, there is a complex bidirectional interaction between hyperlipidemia and gut flora dysbiosis during pregnancy. Studies have noted disrupted gut microbiota in pregnant women with hyperlipidemia. In 2023, research showed lower α‐diversity in dyslipidemic pregnant women, with genera like *Alistipes* and *Bacteroides* negatively correlated with serum triglyceride (TG) levels, potentially reducing dyslipidemia risk. Additionally, the gut microbiota, combined with blood biochemical profile in the second trimester, could predict the risk of dyslipidemia in the third trimester [166]. Furthermore, both animal and human studies have shown that maternal obesity can alter the gut microbiota of both the mother and offspring [167]. On the other hand, gut microbiota dysbiosis can significantly affect lipid metabolism during pregnancy. In hyperlipidemia cohorts during pregnancy, several bacteria were correlated with blood lipid levels, where a lower abundance of *Faecalibacterium* was associated with increased TG levels, and higher levels of *Streptococcus* and *Actinomyces* genera were positively linked to higher total cholesterol (TC) levels [168]. *Faecalibacterium*'s major metabolite, butyric acid, can prevent pregnancy‐induced obesity and hyperlipidemia caused by a high‐fat diet, thereby reducing metabolic disruption [169]. Two meta‐analyses of relevant randomized controlled trials (RCTs) also indicated that probiotic interventions with *Lactobacillus* spp. and *Bifidobacterium* spp. can significantly reduce TC and TG levels and have a substantial effect in reducing the risk of early GDM [170, 171]. Future research and clinical practice should focus on using gut microbiota modulation to treat mild hypertension and dyslipidemia in early to mid‐pregnancy, aiming to lower the risk of related pregnancy complications.

### Sepsis

Sepsis is a fatal multiple‐organ dysfunction caused by an uncontrolled inflammatory response to infection [172]. Pregnancy elevates the risk of developing more severe sepsis, which may lead to serious complications such as PE and stands as a leading cause of maternal morbidity and mortality worldwide [173].

During pregnancy, bacterial infections may arise from various causes including the exist of a specific bacteria, an imbalance in the microbiomes, or the spread of bacteria from the gut. Common causes include *Group B Streptococcus*, from *Streptococcus agalactiae* in the gastrointestinal and genital areas, causing urinary and bladder infections [174]. Additionally, pregnant women and newborns are at a higher risk for listeriosis compared to nonpregnant women [175]. This condition stems from the *L. monocytogenes* bacterium, commonly acquired through the ingestion of food tainted with the pathogen. Both of the mentioned bacterial infections can lead to severe maternal and newborn sepsis [175]. Recent studies have linked maternal sepsis to gut microbiota dysbiosis. For instance, Chen et al. found that transplanting fecal microbiota from pregnant subjects induced an enhanced inflammatory response, hastening mortality in nonpregnant septic mice [176].

Mechanistically, gut dysbiosis during pregnancy reduces *Parabacteroides merdae* and its metabolite formononetin levels. This activates the hnRNPUL2–NLRP3 axis and triggers excessive macrophage pyroptosis in sepsis, impairing bacterial clearance and enhancing inflammation, thereby increasing sepsis susceptibility [176]. This study reveals that microbiome–immune interactions are implicated in the pathogenesis of maternal sepsis, offering new strategies for the precision approach of sepsis in pregnancy.

## ADVERSE PREGNANCY OUTCOMES

### FGR

FGR is defined as the inability of a fetus to achieve its genetic growth potential [177]. These infants are more prone to hypertension, diabetes, obesity, and other diseases in adulthood [178]. The underlying mechanisms and long‐term effects of FGR are not fully understood. Many studies have identified significant gut microbiota alterations between FGR patients and women with normal pregnancies. Specifically, an increased abundance of several butyrate‐producing genera, such as *Bacteroides*, *Faecalibacterium*, and *Lachnospira*, has been observed in FGR patients [179]. A large‐scale study involving 6348 pregnant women revealed a correlation between *Helicobacter pylori* infection and FGR [180]. Yang et al. uncovered that the FGR‐related adverse intrauterine environmental factors had a more significant impact on bacterial diversity and early gut microbiota composition than genetic factors, with this impact positively correlating with the severity of twin FGR [181]. In FGR neonates, they found reduced *Enterococcus* and *Acinetobacter* and lower fecal methionine and cysteine levels, important for physical and neurocognitive development. Surprisingly, this study observed a significantly increased abundance of butyrate producer *Oscillospira* and *Coprococcusin* in twins with FGR [181]. The finding of increased butyric acid‐producing bacteria in mothers and fetuses with FGR challenges the conventional wisdom, as butyrate is generally beneficial for regulating glucose metabolism and reducing inflammation [182]. While many studies report a lack of these bacteria in various diseases, their enrichment in FGR cases suggests a potential compensatory mechanism for intrauterine malnutrition. Additionally, gut *Roseomonas* and Propionibacteriaceae were significantly positively associated with placental and newborn birth weight, whereas *Sphingomonas* and *Marinisporobacter* were significantly negatively correlated with neonatal body mass index and birth weight [183]. According to the metabolism pathway analysis, the functions of the aforementioned differential gut bacteria are closely connected to glucose and nitrogen metabolism [183]. Studies involving FMT and specific dietary interventions suggest an important role for gut microbes in the prevention and treatment of FGR (Figure 3) [184].

### PTB

PTB, defined as childbirth occurring before 37 weeks of gestation [185], is linked to breakdown in maternal–fetal tolerance, vascular diseases, cervical insufficiency, PROM, and intrauterine infections [186]. Studies have linked gut microbiota to PTB. In a prospective cohort study of Japanese mother–child dyads, Shiozaki et al. found significant gut microbiota alterations at 28 weeks gestation in mothers with preterm infants compared to full‐term infants. In the PTB group, *Clostridium* and *Bacteroides* were significantly reduced, while Lactobacillales was markedly elevated [187]. *Clostridia* spp. and *Bacteroides* are potent inducers of Treg cell proliferation and activation. Gut‐derived Treg cells can prevent PTB by suppressing inflammation through IL‐10 production [188]. Hiltunen et al. analyzed the gut microbiota of 55 preterm neonates, 25 full‐term neonates, and 51 mothers and also found that the initial gut microbiota of preterm infants differed significantly from full‐term counterparts [189]. Another prospective cohort study by Dahl et al. observed a reduction in gut microbiota diversity in PTB women, with lower abundance of Clostridiales, *Bifidobacterium*, and *Streptococcus* [53]. Many *Bifidobacterium* species have anti‐inflammatory properties and inhibit LPS‐induced nuclear factor‐κB activation, IL‐8, and cyclooxygenase‐2 production [188]. Therefore, these findings suggest that a decrease in Clostridiales and *Bifidobacterium* may increase susceptibility to inflammation or infection‐induced PTB (Figure 3).

Both FGR and PTB are conditions closely linked to malnutrition. In low‐ and middle‐income countries, maternal flora changes correlate with pregnancy outcomes and birth weight [190]. Infants with FGR are at an increased risk of PTB [191]. As previously mentioned, diet significantly influences the gut microbiota of pregnant women, thereby impacting pregnancy outcomes. For instance, a diet that is poor in quality, low in fiber, and high in fat can decrease the α‐diversity of the maternal gut microbiota, which is related to SPTB [192]. Moreover, levels of docosahexaenoic acid and eicosapentaenoic acid were significantly elevated in the feces of SPTB patients. Therefore, the decreased α‐diversity and the increase in omega‐3 fatty acid levels in the feces of pregnant women might function as predictive biomarkers for SPTB [192].

Until now, the causality between the aforementioned alterations in gut microbiota and FGR and PTB remains unconfirmed. Therefore, future research should conduct longitudinal studies on mothers experiencing FGR and PTB who maintain balanced diets. Systematic examination of probiotic supplementation or FMT from these donors is crucial to assess their microbiota's causal effect on pregnancy outcomes.

## POTENTIAL OF GUT MICROBIOTA IN DIAGNOSING PREGNANCY COMPLICATIONS

Numerous studies have proposed gut microbiota dysbiosis as a potent biomarker for disease. In a prospective study, Israeli researchers developed a model comprising clinical indicators, gut microbiota, and inflammation factors based on the data of 394 first‐trimester pregnant women. This model can accurately predict GDM risk and offers potential for early identification and prevention for GDM, thereby mitigating potential risks to both mother and fetus. Among these parameters, gut microbiota changes, increased serum IL‐6 level, and reduced SCFA and branched SCFA levels in feces are suggested as potential early biomarkers of GDM [193]. Our previous study assessed the diagnostic value of gut microbiota and SCFA in PE. The diagnostic accuracy of PE reached 89.7% based on the abundance of intestinal *Akkermansia* and *Oscillibacter*. When combined with serum propionic and butyric acid levels, systolic and diastolic blood pressure, and urinary protein, the diagnostic efficacy increased to 98.98% [137]. Another study reported an area under the receiver‐operating characteristic curve (AUC) of 0.764 for a diagnostic model of PE based on Kyoto Encyclopedia of Genes and Genomics Orthology of gut microbiota [194]. In addition, SCFAs exhibit diagnostic potential for PE and can serve as diagnostic biomarkers for ICP, with an AUC of 0.968 for hexanoic acid. Isobutyric and valeric acids are potential biomarkers for GDM, with isobutyric acid having an AUC of 0.764 [195]. These gut microbiota‐associated indicators provide a convenient approach for diagnosing pregnancy complications.

However, it is crucial to acknowledge the ongoing debate concerning the value of gut microbiota as disease diagnostic markers. Some studies argue that specific gut bacteria cannot predict the occurrence of pregnancy complications [196]. Furthermore, numerous challenges and limitations exist. For instance, inconsistencies in gut bacterial profiles of the same disease across studies raise questions about their diagnostic reliability. Moreover, factors such as individual variation, geographical location, dietary habits, and ethnicity significantly influence gut microbiota. Consequently, gut microbiota markers identified in small, region‐specific studies may not be universally applicable. However, to date, only a few meta‐analyses and small‐sample studies have explored the generalizability of healthy gut microbiota baselines and disease models across different geographical populations. In addition to factors such as location and diet, differences in sample collection, preparation, sequencing, and data analysis methods contribute to significant variability in results.

Therefore, to establish a reliable diagnostic model for pregnancy complications based on changes in gut microbiota, the above‐mentioned issues must be addressed. Solutions could involve regionalized studies, extensive sampling, standardized experimental protocols, feasibility screening of gut microbial markers (eliminating gut bacteria known to be susceptible to factors such as diet and geography and selecting those with relatively stable abundance in population), and combination with other clinical indices. Additionally, for diseases with reliable diagnostic indicators, the focus should shift toward evaluating the potential of gut microbiota in early detection and prognostic prediction.

## POTENTIAL OF GUT MICROECOLOGICAL THERAPY FOR PREGNANCY COMPLICATIONS

### Probiotics

**Figure 5:**
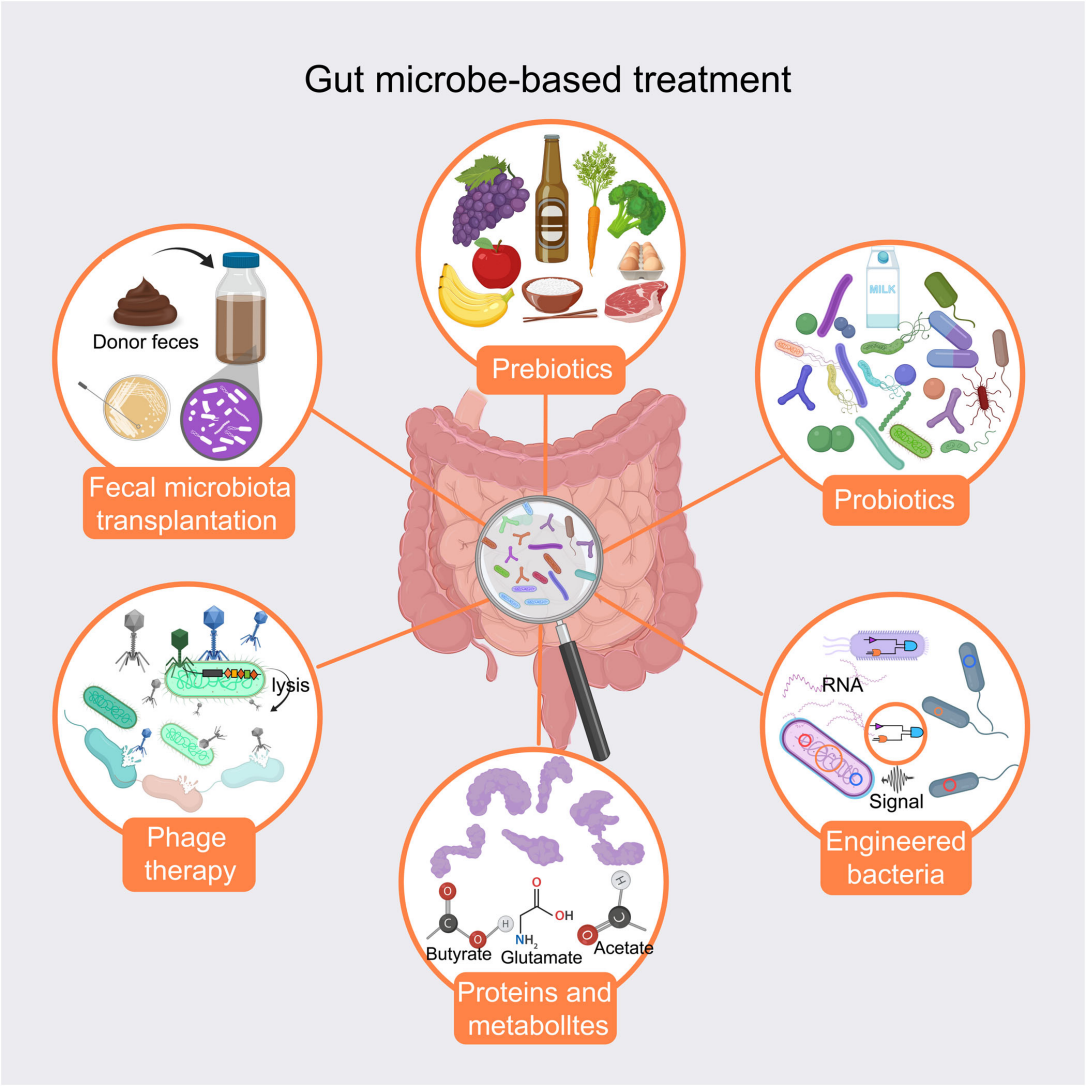
Potential gut microbe‐based treatment for pregnancy complications. Schematic illustration of current potential strategies to improve pregnancy complications by manipulating the gut microbiota, including prebiotics, probiotics, fecal microbiota transplantation, phage therapy, engineered bacteria, and proteins and metabolites.

Probiotics, active microorganisms beneficial to health when consumed in adequate amounts, have been used to treat pregnancy complications (Figure 5) [197]. Daily administration of *Lactobacillus acidophilus*, *Lactobacillus casei*, *L. fermentum*, and *Bifidobacterium bifidum* during mid‐pregnancy can significantly ameliorate insulin resistance and dysregulated lipid metabolism in GDM patients [198, 199]. A correlation has been observed between the intake of lactic acid bacteria products and a decreased incidence of PE [200]. However, the effectiveness of probiotics is debated. A double‐blind RCT showed that even if used from the early phase of the second trimester, probiotics were ineffective in preventing GDM among overweight and obese pregnant women [201]. Several meta‐analyses also hinted at the lack of substantial evidence supporting the significant impact of probiotic intake during pregnancy on fasting plasma glucose, PTB risk, or any other adverse maternal or neonatal outcomes [202, 203, 204]. These conflicting results may be attributed to discrepancies in the species of probiotics used, the duration and dosage of treatment, and the severity of the patients' conditions across different studies, underscoring the necessity for standardization of experimental factors in gut microbiota research.

### Prebiotics

#### Prebiotics and pregnancy complications

Prebiotics, a class of dietary fibers not easily digested or absorbed by humans but beneficial for gut bacteria growth and activity, are gaining attention for their potential to treat and prevent pregnancy complications (Figure 5). Several studies suggest prebiotics may reduce GDM, hypertension, and other complications by fostering a healthy gut microbiome and reducing inflammation [205]. Prebiotic supplementation during pregnancy, such as oligogalactose, can modify gut microbiota, increasing Bacteroidetes and SCFAs, decreasing Firmicutes, and creating an immune‐tolerant uterine environment. This can lower allergy and autoimmune disease risks in offspring [206]. Maternal obesity, a risk factor for offspring obesity, may be mitigated by prebiotics like polydextrose, restoring gut microbiome balance and enhancing skeletal muscle mitochondrial function in offspring [207]. Dietary modifications exert positive effects on the homeostasis of host gut microbiota. The intake of high‐fiber or high‐protein diets during pregnancy and lactation differentially affects the expression of satiety hormones and genes related to glucose and lipid metabolism in offspring, thereby influencing the risk of obesity, insulin resistance, and other metabolic disorders [208]. Additionally, the inclusion of free amino acids in diets can influence the composition and metabolic functions of microbiota, which in turn has implications for the health of the host. This suggests that AAs could be considered a new type of prebiotic, potentially labeled as “aminobiotics” [209]. Nevertheless, further research is necessary to confirm these findings and to determine optimal dosage and timing of prebiotic supplementation to effectively prevent pregnancy complications. While generally safe, prebiotics may cause bloating and other gastrointestinal side effects.

#### Traditional Chinese medicine (TCM)

Many traditional Chinese herbs rich in bioactive compounds, proteins, and polysaccharides, are considered prebiotic and significantly impact gut microbiota, potentially explaining their therapeutic effects [210].

Gan Cao, scientifically known as *Glycyrrhiza uralensis* Fisch., contains the primary component diammonium glycyrrhizinate, which can effectively regulate the gut microbiota. It reduces endotoxin‐producing bacteria such as *Desulfovibrio* and increases SCFA‐producing probiotics such as *Lactobacillus* and Ruminococcaceae. Such restoration of gut microbiota helps protect mice from nonalcoholic fatty liver disease [211]. Given that patients with GDM often have increased *Desulfovibrio* and decreased *Lactobacillus* in the gut, Gan Cao might counter these detrimental changes, offering potential benefits for GDM treatment [121, 122].

Huang Qi, scientifically known as *Astragalus membranaceus*, has emerged as a notable candidate for type 2 diabetes mellitus (T2DM) treatment. Recent research demonstrates that Huang Qi effectively rectifies disorders in glucose and lipid metabolism in streptozotocin‐induced T2DM mice, mitigates inflammation and oxidative stress, and minimizes organ damage. Its hypoglycemic and hypolipidemic properties may be ascribed to its capacity to remodel the specific gut microbiome. Specifically, Huang Qi suppresses intestinal pathogens like *Shigella* and boosts beneficial bacteria such as *Allobaculum* and *Lactobacillus*, aiding gut barrier repair [212]. Furthermore, Huang Qi has been found to significantly alleviate diabetes symptoms in db/db model mice, concurrently restoring the balance of their gut microbial community and enhancing SCFA levels. The rise in gut bacteria like *Akkermansia* and *Bacteroides* correlates positively with its hypoglycemic effects and the production of SCFAs in the feces. Mechanistically, the SCFA boost induced by Huang Qi upregulates GPR41/43 and tight junction proteins like occludin and zona occludens 1, stimulates GLP‐1 secretion, and improves intestinal integrity, thus effectively alleviating diabetes symptoms in mice [213]. These investigations elucidate the intricate interplay between Huang Qi‐regulated gut microbiota and its effects on glucose and lipid metabolism.

Dan Shen, identified scientifically as *Salvia miltiorrhiza*, has been shown to markedly enhance the structure of gut microbiota, specifically by increasing the abundance of *Akkermansia*, *A. muciniphila*, and *Lactobacillus intestinalis* in SHR rats. These microbiotic alterations are potentially linked to Dan Shen's multifaceted therapeutic roles, including its anti‐inflammatory, antiplatelet aggregation, vasodilatory, and antioxidative stress activities [214]. Intriguingly, in patients with PE, there is a notable decrease in the abundance of *Akkermansia* and *A. muciniphila* in the gut [137]. This observation suggests a potential preventive role for Dan Shen in the context of PE.

Ling Zhi (*Ganoderma lucidum*)'s water extract has a significant effect against obesity, maintaining gut barrier integrity, reducing ME, and lowering the expression and secretion of proinflammatory cytokines (such as TNF, IL‐1β, and IL‐6). It also improves glucose tolerance and insulin sensitivity, and these effects are closely related to its regulation of the gut microbiota. For example, transferring fecal microbiota from Ling Zhi‐treated mice to those on a high‐fat diet replicates the weight loss effect of Ling Zhi [210]. These insights highlight the vital role of gut microbiota regulation in the effectiveness of these TCM treatments.

Currently, there are no reported studies on the relationship between TCM, gut microbiota, and pregnancy complications. However, the mentioned literature already demonstrates the importance of gut microbiota as intermediaries in the regulation of glucose, lipids, and blood pressure by TCM, suggesting a promising potential for TCM in treating pregnancy complications by modulating gut microbiota.

#### Fermented foods and beverages

Fermented foods and beverages can be defined as foods made through desired microbial growth and enzymatic conversions of food components [215]. Typical probiotic‐rich fermented foods often encompass yogurt, kefir, kombucha, sauerkraut, pickles, miso, tempeh, kimchi, sourdough bread, and various cheeses. Yogurt, as a popular probiotic food, typically contains probiotic counts ranging from 90 to 500 billion colony‐forming units (CFUs) per serving. The health benefits of these foods arise from the probiotics they contain, the transformation of elements like biologically active peptides, biogenic amines, and phenolic compounds during fermentation, and the reduction of nutrient absorption inhibitors.

The effects of certain fermented foods on gut health, such as kefir, sauerkraut, natto, and sourdough bread, have been examined in RCTs. Kefir, in particular, a well‐studied fermented product, is scientifically recognized for significantly improving lactose intolerance and aiding in *H. pylori* eradication [215, 216]. However, broader clinical evidence on the effects of most fermented foods on gastrointestinal health and disease prevention remains limited.

Existing research has explored the cardioprotective effects of fermented dairy products containing live microorganisms [217]. Meta‐analyses of prospective studies suggest a moderate inverse relationship between overall dairy consumption and cardiovascular disease (CVD) risk. However, findings on fermented dairy products like yogurt and cheese are inconsistent, leading to ongoing debates among researchers [218]. In contrast, numerous studies have shown the benefits of fermented foods and beverages without live microorganisms in CVD prevention [217].

According to the above results, the impact of fermented foods and beverages on human health remains uncertain; further cohort studies and RCTs are essential to verify their effects on various diseases. However, growing studies on fermented foods in gastrointestinal and CVD contexts show promise in reducing blood pressure and cholesterol levels, offering insights for potential use in managing pregnancy‐related complications.

Research is relatively scarce in the field of maternal and child health. A new research indicates that a traditional fermented food known as injera, commonly made in Ethiopia, might play a role in enhancing the diversity of gut bacteria in Ethiopian infants. These bacteria, not typically found in the human microbiome, are likely introduced into the infants' system through the consumption of such fermented foods [219]. This suggests the potential of fermented foods in shaping the gut microbiota of both mothers and infants.

#### RCTs of prebiotics

RCTs are crucial for determining the actual effectiveness of prebiotics. In 12 out of 15 RCTs, adding prebiotics like galacto‐oligosaccharide (GOS) and fructo‐oligosaccharide (FOS) to infant formula (IF) has led to increased fecal *Bifidobacterium* levels [220]. A recent RCT showed that GOS intervention modified the gut microbiota in pregnant women without impacting glycolipid metabolism and clinical indices [221]. Therefore, the dietary supplementation of prebiotics during pregnancy requires further clinical investigation. Future studies should maintain consistency in microbial assessments and thoroughly document the composition of solid foods introduced during interventions.

### Synbiotics

Synbiotics, a combination of prebiotics and probiotics (Figure 5), boost beneficial gut bacteria growth and help maintain a healthy gut microbiota [222]. A synbiotic capsule containing *L. acidophilus* (5 × 10^10^ CFU/g), *Lactobacillus plantarum* (1.5 × 10^10^ CFU/g), *Lactobacillus fermentum* (7 × 10^9^ CFU/g), *Lactobacillus gasseri* (2 × 10^10^ CFU/g), and 38.5 mg of FOS, when administered for 6 weeks to GDM patients, can significantly ameliorate oxidative stress and abnormal lipid metabolism without affecting insulin sensitivity [223]. Conversely, another synbiotic with inulin alleviated insulin resistance and oxidative stress [224]. Additionally, dietary intervention with fish oil and probiotics can increase the abundance of *Bifidobacterium* and decrease the abundance of *Haemophilus parainfluenzae*, *Veillonella parvula*, and *Eggerthella lenta* in the gut microbiota of healthy pregnant women [196]. These effects might be due to the promotion of adherence and colonization of probiotic bacteria in the gut by fish oil [225]. However, some studies found no reduction in GDM incidence with fish oil and probiotics [196]. These contradictory results emphasize the need for further large‐scale clinical studies to better understand the role and therapeutic value of synbiotics in pregnancy complications.

### FMT

FMT involves transplanting fecal matter from a healthy donor into a patient's gastrointestinal tract (Figure 5). It has been successful in treating intestinal disorders like *Clostridium difficile* infection, IBD, and irritable bowel syndrome [226, 227]. Additionally, newborns delivered by cesarean section can re‐establish their gut microbiota through FMT using postnatal maternal microbiota [228]. Nonetheless, the potential of FMT in treating and preventing pregnancy complications remains underexplored to date. Although FMT offers a broader spectrum of probiotics compared to probiotic therapy, challenges include obtaining healthy donors, unstable efficacy, the high risk of transmitting infections, and unknown long‐term effects on pregnant women and newborns. Increasing evidence suggests that an advanced technique known as WMT, which employs microfiltration to eliminate fecal particles, parasite eggs, and fungi during the preparation of samples, offers a more accurate, manageable, and secure method compared to traditional FMT [229]. Studies have shown that WMT sourced from healthy individuals can effectively lower blood pressure in hypertensive patients [230].

Therefore, in light of the uniqueness of pregnant women and fetuses, we recommend that future research should focus on the following aspects: First, the use of rapid and precise metagenomic sequencing methods to thoroughly assess the infection risk of donors and their compatibility with the recipient's microbiota. Second, the optimization of the preparation, delivery, and storage methods for FMT, while eliminating the influence of toxins and other hazardous substances, to minimize potential risks to both the mother and infant. Third, conducting comparative analyses between FMT and alternative approaches such as WMT. Lastly, conducting more multicenter, double‐blind RCTs to assess the safety and efficacy of FMT during pregnancy, especially among pregnant women in different gestational periods and with diverse backgrounds.

### Phage therapy

Recent animal studies suggest that broad‐spectrum antibiotics can adversely affect fetal health by disrupting gut microbiota and metabolic pathways [231]. They also negatively impact neurodevelopment, including reduced neuron numbers in the hippocampus, smaller astrocytes, impaired neuron growth in the dentate gyrus, and loss of hippocampal synapses, potentially affecting early cognitive development in children [232]. Moreover, antibiotic treatment in pregnant mice can alter immune cell distribution, like increasing splenic Th1 cells and activated blood monocytes, while reducing Th2, Th17, and double‐positive FoxP3/RoRgT cells in the Peyer's patches and mesenteric lymph nodes, disrupting maternal immune regulation [233]. Early childhood exposure to certain broad‐spectrum antibiotics may increase the risk of overweight, though this link does not persist into later childhood [234]. Given these findings, assessing the risks and benefits of antibiotic therapy during pregnancy is crucial. The US Food and Drug Administration (FDA) published the “Pregnancy and Lactation Labeling Rule” to provide detailed guidance for healthcare professionals and patients about the use of prescription drugs and biologics during pregnancy and lactation, helping to understand drug safety [235, 236]. Antibiotics indiscriminately kill both beneficial and harmful bacteria, and their overuse can lead to the emergence of drug‐resistant strains.

Facing the growing problem of bacterial resistance and the rise of superbugs, the medical field is re‐evaluating phage therapy—a treatment that can precisely target specific host bacteria without disrupting the overall microbial balance. In 2020, a patient with sepsis from diabetic foot ulcers was treated unsuccessfully with the heaviest hitting cocktails in the antibacterial arsenal for a multidrug‐resistant infection. As a final measure, doctors chose to administer over one billion viral particles intravenously, which improved his condition and cleared the carbapenem‐resistant *Acinetobacter baumannii* respiratory infection [237]. Phage therapy, as a complement to antibiotic treatment, offers a precise antibacterial strategy and brings new hope for combating drug‐resistant infections (Figure 5). Therefore, the use of antibiotics during pregnancy should be approached with caution, considering their type, mechanism of action, genetic toxicity, safety during pregnancy, and potential long‐term adverse effects on the fetus [238]. While antibiotics have a significant impact on the gut microbiota, they should be used judiciously as a treatment option for pregnancy complications. Alternative and more precise methods, such as probiotics, prebiotics, and phage therapy, should be considered first.

### Clinical trials of intestinal microecological therapy

Clinical trials for gut microecological therapy in pregnancy complications are limited, but promising results have emerged in other diseases. A 2021 phase 2 RCT revealed that a microbiota‐directed complementary food prototype (MDCF‐2) was more effective in improving weight‐for‐length and weight‐for‐age in young children with moderate acute malnutrition than standard ready‐to‐use food, highlighting its potential as a dietary supplement [239]. Additionally, three randomized, double‐blind, placebo‐controlled clinical trials demonstrated the therapeutic potential of RBX2660 (a fecal microbiota suspension) [240], SER‐109 (a combination of dozens of microbiota) [241], and VE303 (eight *Clostridium* strains) [242], which significantly reduced or prevented the recurrence of *C. difficile* infection with no adverse events.

In clinical trials, various intestinal microecological intervention approaches have their pros and cons. Dietary modifications and prebiotics are safe and easily prepared, yet the persistence of their effects and the specificity of targeted symbiotic bacterial strains still require further evaluation. Regarding symbiotic microbial consortia, they can screen the safety of known flora compositions and individual isolates. However, there are challenges in strain selection and cultivation, including potential mutations during cultivation, maintenance of bacterial functionality after long‐term cultivation, unexpected phenotypes from complex bacterial interactions, and the need to ensure phenotype, homogeneity, purity, and efficacy of the used bacteria. As for FMT, which integrates the benefits of an entire microbial community, concerns remain about the transmission of pathogens and virulence, as well as the variable efficacy that is heavily dependent on donor selection [243]. The determination of which methods will be widely adopted in the future is still an open question in the field.

Developing new therapies, especially live bacterial drugs, is a complex and costly process that involves many steps from strain cultivation to FDA approval. While preclinical studies can be conducted in standard laboratories, clinical trials of live bacteria must adhere to strict manufacturing regulations and require extensive and iterative studies of various living biological agents. These difficulties limit the development of novel therapies. Currently, these developments are primarily carried out by commercial entities [243].

To overcome these challenges, increased support from nonprofit organizations, such as governments, foundations, and charities, is needed. Furthermore, it is worth considering innovative research and development (R&D) models, such as public–private partnerships, which combine the implementation capabilities of commercial firms with the research capacities and resources of public organizations (e.g., laboratories), thereby enhancing R&D efficiency.

## THE DEBATE ON “STERILE WOMB” AND “IN UTERO COLONIZATION”

### Sterile womb

Historically, it was believed that fetuses develop in a sterile environment. In 1885, Theodor Escherich reported no viable microbes in meconium [244]. Subsequent studies in 1927 and 1934 demonstrated negative bacterial cultures in meconium samples from 62% of healthy pregnant women [245, 246]. Another study in 1927 found that all amniotic fluid from women who had been in labor for less than 6 h tested negative for bacteria, while that from women who had been in labor for more than 6 h tested positive [247]. Subsequent research largely upheld the sterile uterus concept [248, 249, 250]. Although some research has reported a significant increase in the bacterial positivity rate of meconium in pregnancy complications, such as PROM [251, 252, 253], PTB [254, 255], PE [256], and FGR [257], a total of 68% of meconium samples still tested negative for bacteria even when combining culture, PCR, and sequencing techniques. Further, a 2016 study found that the bacterial DNA isolated from placental samples was indistinguishable from the bacterial DNA present in DNA extraction kits, and its abundance was too low to determine whether it was a true signal or contamination [258]. A comprehensive 2019 study mostly attributed positive bacterial signals in placental samples to contamination, although *Streptococcus lactis*, a neonatal sepsis pathogen, was identified in 5% of the predelivery samples [259]. In 2023, a study published in *Nature* conducted a thorough analysis of the data from prominent studies by Rackaityte et al. and Mishra et al. (as mentioned in the “In utero colonization” section). This study refuted the concept of utero colonization. It concluded that microbial signals in fetal samples likely result from contamination during clinical procedures or DNA extraction and sequencing. Furthermore, live microbes thriving in healthy fetal tissues contradict fundamental concepts of immunology, clinical microbiology, and the generation of germ‐free animals [260]. On the other hand, some researchers believe that the so‐called “placental microbiota” might originate from the vagina, as some bacteria identified in the placenta are identical to those in the vagina. In 2021, Sterpu et al. also found that more bacteria could be cultured from the placentas following vaginal delivery than from cesarean delivery [261]. All in all, these studies suggest that while the placenta may harbor pathogens, it is highly unlikely for a stable bacterial community to be pervasive in a normal placenta, implying that a healthy uterus is sterile. If bacteria are detected, it is more likely due to contamination during the delivery process or in the laboratory environment (Table 1).

**Table 1:** Timeline of the “sterile womb” hypothesis.

Year	Sterile womb	References
1885	No viable bacteria was detected in the meconium	[244]
1927	Amniotic fluid bacterial culture: labor under 6 h (−), labor over 6 h (+)	[247]
1927–1934	Cultures of meconium samples are bacteria‐free	[245, 246]
1962–1976	Sterile amniotic cavity: positive cultures attributed to collection contamination	[248, 249, 250]
1982	Bacteria in meconium, placental and amniotic fluid: (−) in healthy cohort; (+) in partly PROM patients via Gram stain, IF and culture	[251]
1986–1990	Echoing 1982 findings: Bacteria (−) in healthy cohort; (+) in part of PROM patients	[252, 253]
2008–2013	Despite the detection of bacteria (+) in various pregnancy complications such as PTB, PE, and FGR via methods like PCR, staining, or culture, the total detection rate remains low	[254, 255, 256, 257]
2016	Low abundance of bacterial DNA sequencing in the placenta is linked to contamination from extraction kits	[258]
2019	No microbiome in placental samples; most signals are from labor/delivery or lab contamination	[259]
2021	16S rRNA sequencing cannot distinguish between bacteria in the placenta and lab contaminants; skin and vaginal bacteria predominate in placental cultures.	[261]
2023	Microbial signals could be due to contamination from clinical handling or DNA processing steps	[260]

Abbreviations: FGR, fetal growth restriction; IF, immunofluorescence; PCR, polymerase chain reaction; PE, pre‐eclampsia; PROM, premature rupture of membranes; PTB, preterm birth; rRNA, ribosomal RNA.

### In utero colonization

Recent DNA sequencing advancements have enabled the detection of previously unculturable microorganisms. In 2013, Indira Mysorekar found that nearly one‐third of 200 placenta sections examined under a microscope contained bacteria [262]. In a representative study conducted in 2014, researchers sequenced the whole genomes of placentas from over 300 women under sterile conditions, uncovering a sparse bacterial community in the placenta resembling oral bacteria, different from those in other body parts [263]. This finding challenges the conventional view that neonates acquire their microbiota through birth, suggesting instead that oral bacteria might enter the placenta via the bloodstream. Since then, numerous studies have reported the possibility of microbial presence in the placenta. For instance, Gomez‐Arango et al. supported the “oral origin” hypothesis of the infant microbiome and suggested a gut microbiome contribution [264]. Other research indicates that placental bacterial signatures are independent of delivery method and contamination during delivery, suggesting prebirth bacterial presence [265]. Parnell et al. further demonstrated the spatial heterogeneity in the distribution of microbial communities within the placenta [266]. In addition, they used various methods, including Warthin–Starry staining, Gram staining, in situ hybridization of bacterial ribosomal RNA (rRNA), fluorescence in situ hybridization (FISH), and clinical culture, and confirmed the presence of microbes in the placenta [267, 268]. All these studies indicate bacterial colonization in the uterus during a healthy pregnancy, although with extremely low abundance and diversity.

Recently, Rackaityte et al. identified small bacterial populations in meconium, including Micrococcaceae and *Lactobacillus*, using 16S rRNA sequencing, scanning electron microscopy (SEM), and FISH. They further isolated *Micrococcus luteus*, a bacterium that thrives in fetal intestine‐like environments and enhances the recruitment of immune cells [269]. Mishra et al. subsequently corroborated the widespread presence of *Lactobacillus* and *Staphylococcus* in various tissues. They also reported T‐cell activation in fetal tissues, suggesting antigenic stimulation before birth. These findings strongly support in utero bacteria presence and potential fetal immune system priming [270]. In 2022, La et al. used 16S sequencing to suggest that the placenta might contain trace amounts of microbiota, with notable variations in its dominant species linked to different adverse pregnancy outcomes [271]. In 2023, Stupak et al. detected bacterial proteins in placentas with late FGR and controls using liquid chromatography‐electrospray ionization‐tandem mass spectrometry technology [272]. A review article published in *Gut* in 2023 also argued that the initial encounters with microbes may already occur in the womb, preparing for interaction with a bacteria‐rich environment at the time of delivery [273]. Nevertheless, contamination in low biomass samples such as placenta, uterus, and fetal tissue remains an unavoidable and urgent problem (Table 2).

**Table 2:** Timeline of the “in utero colonization” hypothesis.

**Year**	**In utero colonization**	**Reference**
2013	Morphological indications of bacteria in 26% of healthy deliveries, higher in preterm: Evidence from placental basal plate paraffin sections	[262]
2014	16S rRNA sequencing and WGS metagenomic studies reveal a unique placental microbiome that closely resembles the human oral microbiome	[263]
2017	(1) Placental microbes mirror oral microbes, with both oral and gut sources seeding the placenta, suggesting diverse origins for placental colonization and (2) the placental microbiota exhibits niche specificity	[264, 266]
2019	16S rRNA sequencing, FISH, staining, and culture reveal bacterial presence in utero compartment and fetal Intestine	[267, 268]
2020	16S rRNA sequencing, SEM, and FISH reveal a limited microbiota in fetal meconium, dominated by Micrococcaceae and *Lactobacillus*. *Micrococcus luteus* is isolated, exhibiting unique growth patterns in in utero‐like intestinal conditions	[269]
2021	*Staphylococcus* and *Lactobacillus* in fetal tissues activate memory T cells in fetal mesenteric lymph nodes, demonstrating the importance of microbial exposure in fetal immune priming	[270]
**2022**	16S rRNA sequencing shows the placenta has trace microbiota, varying with pregnancy complications	[271]
**2023**	LC‐ESI‐MS/MS technology detected many bacterial proteins in placentas from both healthy controls and pregnancies with late FGR	[272]

Abbreviations: FISH, fluorescence in situ hybridization; LC‐ESI‐MS/MS, liquid chromatography‐electrospray ionization‐tandem mass spectrometry; rRNA, ribosomal RNA; SEM, scanning electron microscopy; WGS, whole‐genome sequencing.

### Solution

Despite growing evidence suggesting the existence of uterine microbes, concerns about experimental contamination persist. Addressing this issue is critical for elucidating the roles of gut microbiota in the regulation of uteroplacental function and in the pathogenesis of pregnancy complications, as well as for determining the timeline of microbiome origin in newborns.

Given that various pregnancy complications are associated with infections, we therefore propose several recommendations: (1) Strict adherence to aseptic practices. Minimizing contamination during sample collection, transportation, and handling is paramount. Possible strategies include collecting samples from within tissues and using sterile tools and equipment; (2) use of appropriate controls in analyses. Sterile water and placentas from germ‐free animals can serve as negative controls, while samples from confirmed cases of uterine infection can serve as positive controls; (3) ensure all samples are subjected to the same experimental procedure. Employing multiple kits or sequencing companies is essential to determine the inherent background noise of the detection system, thereby reducing the false positive rate; (4) combine culture‐independent techniques (e.g., 16S rRNA gene sequencing, metagenomic sequencing, FISH, histological methods, SEM) with culture‐dependent techniques for bacterial identification and activity assessment; and (5) ascertaining the true origin of intrauterine microbiota, which could be natural or a result of diseases like endometritis or bacterial vaginosis. Are they derived from the evolution of life? Are they a result of the translocation of oral or gut microbiota via the bloodstream or ascending from the vagina? Is the detected signal simply from bacterial fragments? Do bacteria truly colonize the uterus or only reside transiently?

In summary, whether a healthy uterus harbors a microbial community remains an unresolved question. If microbes are indeed present, understanding their origins, the timeline of colonization, and functional characteristics becomes essential. Answering these questions is crucial for determining whether microbes directly influence maternal health through their structural components, such as flagella, or indirectly through their metabolites in the bloodstream. It is also pivotal for elucidating the origins of the fetal microbiome and immune system.

## OTHER NONBACTERIAL GUT MICROBIOTA AND PREGNANCY COMPLICATIONS

Metagenomic sequencing technologies have enabled the examination of nonbacterial microbiota in the human gut, especially gut viruses and fungi, apart from bacteria [274]. Studies have reported changes in the abundance of certain viruses associated with GDM in neonatal meconium. Gut fungi are also suspected to be intricately related to the metabolic status of patients with GDM. For instance, *Penicillium*, *Ganoderma*, *Fusarium*, *Chaetomium*, and *Heterobasidion* are significantly enriched in healthy pregnant women, whereas *Hanseniaspora*, *Torulaspora*, *Auricularia*, *Alternaria*, and *Candida* are significantly enriched in patients with GDM. Moreover, a significant negative relationship between blood glucose levels and intestinal *Ganoderma* has been found [275]. Nonetheless, compared to gut microbiota, our understanding of the changes, roles, and mechanisms of gut viruses and fungi in pregnancy complications remains largely uncharted. Surprisingly, current research on the virome is beginning to emerge, and fecal virome transplantation (FVT) has been found to be capable of altering the gut microbiota, improving glucose tolerance and obesity phenotypes [276, 277]. Transferring fecal virome enhances the proliferation of *A. muciniphila* in the gut and, surprisingly, increases the fertility rate in mice [278]. Therefore, future efforts should focus on exploring the versatile roles and therapeutic mechanisms of viruses, fungi, and their combinations in various pregnancy complications. Aiming to effectively combine these elements for advanced FMT and microbiota‐based therapies is a novel and promising prospect.

Additionally, we recommend the following directions for future research in gut microbiota studies: (1) Utilize a multiomics approach, combining metagenomics, metaviromics, metatranscriptomics, and metaproteomics, along with single‐bacteria‐level sequencing methods, to investigate interactions within and between different bacterial/viral species, as well as their mutual regulation with the host [279, 280]; (2) develop high‐throughput experimental methods that can efficiently and accurately identify the host bacteria of phages; (3) combine FVT and FMT to uncover causal relationships between abnormal gut viromes, gut bacteria, and diseases and to reveal the mutual regulation and mechanisms between gut viruses/phages and the gut microbiota; (4) employ various functional research methods to clarify the roles and mechanisms of specific phages and their host bacteria in the development of diseases, both in vitro and in vivo, and assess their value in disease diagnosis and treatment.

## OUTLOOK

Accumulating evidence reveals the pivotal roles of gut microbiota in human health. Particularly during pregnancy, the species composition of gut microbiota and its metabolites (especially SCFAs) undergo significant dynamic changes as pregnancy progresses, potentially forming part of the physiological adaptation to gestation. Thus, gut microbiota dysbiosis plays a crucial role in the onset of pregnancy complications. These abnormalities provide unique and valuable insights for the diagnosis, early detection, and prognostic evaluation of pregnancy complications. Furthermore, focusing on gut microbe‐based interventions, such as probiotics, prebiotics, synbiotics, FMT, WMT, and FVT, offers new options for treating pregnancy complications.

However, numerous factors, such as sample size, diet, geography, ethnicity, study methodology, and the complexity of disease pathogenesis, significantly influence our understanding gut microbiota's role in pregnancy complications, leading to ongoing debate about its exact functions. Some studies even report that interventions targeting gut microbiota have no significant improvement in the incidence or prognosis of pregnancy complications. To address these issues, it is necessary to carry out large‐scale RCTs to effectively control potential confounding factors. This approach will help clarify the relationship between gut microbiota and pregnancy complications and their potential diagnostic and therapeutic value.

## AUTHOR CONTRIBUTIONS

Qunye Zhang offered valuable insights and supervision during the preparation of this manuscript. Zhenyu Tian wrote the manuscript. Zhenyu Tian and Qunye Zhang revised the manuscript. Xinjie Zhang, Guixiang Yao, Jiajia Jin, Tongxue Zhang, Chunhua Sun, and Zhe Wang critically evaluated and provided substantial suggestions for the manuscript. All authors have reviewed the final version and approved it for publication.

## CONFLICT OF INTEREST STATEMENT

The authors declare no conflict of interest.

## Supporting information


**Table S1**: Comprehensive list of β‐glucuronidases (GUS) sequences identified by Pollet et al. 2017 (DOI: 10.1016/j.str.2017.05.003) and reanalyzed by Candeliere et al. 2022 (DOI: 10.3389/fmicb.2022.826994).
**Table S2**: List of representative LPS‐producing bacteria within the Proteobacteria phylum.

## Data Availability

This manuscript does not generate any code or data. Supporting information (tables, graphical abstract, slides, videos, Chinese translated version, and updated materials) may be found in the online DOI or iMeta Science http://www.imeta.science/.
